# Taphonomic analysis at Liang Bua reveals the behavioral and technological capabilities of *Homo floresiensis*

**DOI:** 10.1126/sciadv.aeb7219

**Published:** 2026-07-03

**Authors:** E. Grace Veatch, Nico Alamsyah, Michael Pante, Alex Pelissero, Tewabe Negash, Briana Pobiner, Chelsea R. Betts, Jatmiko, Thomas Sutikna, Matthew W. Tocheri

**Affiliations:** ^1^ Human Origins Program, Department of Anthropology, National Museum of Natural History, Smithsonian Institution, Washington, DC, USA.; ^2^ Cluster of Excellence in Human Origins, University of Tübingen, Tübingen, Germany.; ^3^ Pusat Riset Arkeometri, Badan Riset dan Inovasi Nasional, Jakarta, Indonesia.; ^4^ Department of Anthropology & Geography, Colorado State University, Fort Collins, CO, USA.; ^5^ Department of Anthropology, University of Connecticut, Storrs, CT, USA.; ^6^ Pusat Riset Arkeologi Prasejarah dan Sejarah, Badan Riset dan Inovasi Nasional, Jakarta, Indonesia.; ^7^ Australian Research Council Centre of Excellence for Australian Biodiversity and Heritage, University of Wollongong, Wollongong, Australia.; ^8^ Department of Anthropology, Lakehead University, Thunder Bay, Canada.

## Abstract

Since its discovery, *Homo floresiensis*—an extinct, short-statured, and small-brained hominin species from Flores, Indonesia—has often been ascribed unexpectedly advanced behaviors, such as hunting large game and using fire. Here, we report the results of a systematic taphonomic study sampling the proboscidean bone assemblage at Liang Bua where the frequency and locations of predatory marks, along with skeletal part profiles, show that Komodo dragons likely had primary access to these remains leaving behind only low-utility elements for *H. floresiensis* to scavenge. Moreover, no signs of intentional use of fire are present in the stratigraphic units associated with *H. floresiensis*. Together, these results suggest that *H. floresiensis* was not as behaviorally advanced as originally suggested and provides critical insights into the behavioral repertoire of *H. floresiensis*, raising important questions about its ancestry.

## Teaser

*Homo floresiensis* scavenged *Stegodon* and did not use fire, challenging previous claims of advanced behavior.

## INTRODUCTION

**Fig. 1.**
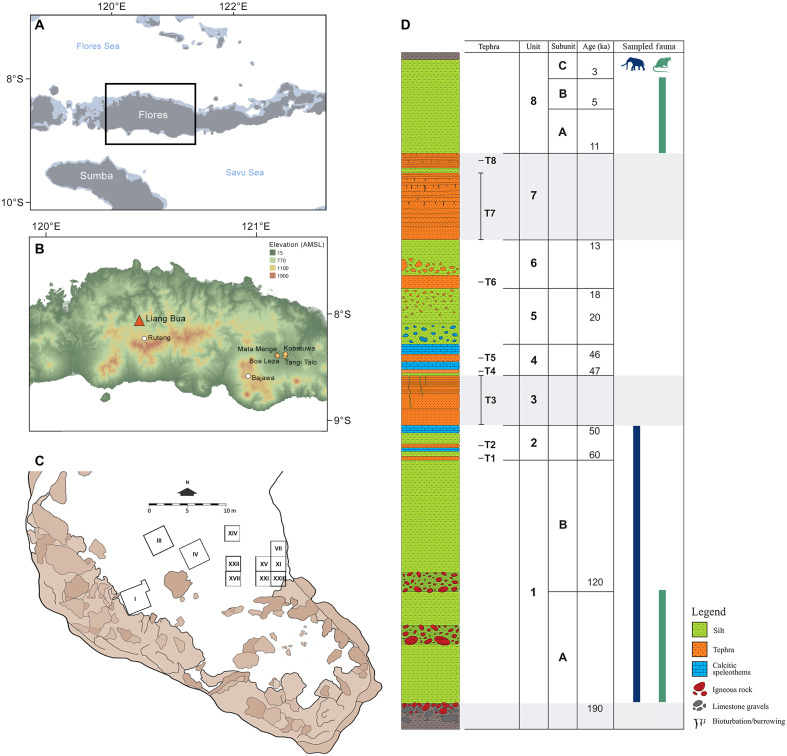
Site location and schematic of the stratigraphy from Liang Bua. (**A**) Location of Flores within the Indonesian archipelago (gray: current coastal land masses; blue: coastlines during the last glacial maximum). (**B**) Location of modern towns (white circles), Liang Bua (red triangle), and other sites (yellow circles) on Flores that have yielded *Stegodon* remains. Maps generated using QGIS [QGIS Development Team (*74*)]. (**C**) Site plan of excavated sectors that yielded the skeletal samples used in this study. (**D**) Stratigraphic composite with associated age ranges modified from (*13*). Subunits from which murine (green) and *Stegodon* (blue) remains were sampled in this study are delineated on the right.

*Homo floresiensis* was originally described as having relatively advanced behaviors for a short-statured and small-brained hominin species based on purported evidence of fire use and the hunting of large game (*1*–*3*). For example, skeletal remains of *H. floresiensis* and a dwarfed species of proboscidean (*Stegodon florensis insularis*) were uncovered together at Liang Bua in association with dense concentrations of stone artifacts, interpreted at the time of discovery as “big game” hunting technology (*2*, *3*) (Fig. 1). This interpretation was further amplified by evidence of cutmarks reported on three *Stegodon* bones (*4*, *5*) and endocast reconstructions of *H. floresiensis* suggesting an unusual expansion in the frontal polar region associated with higher cognitive processing (*6*). Some of the smaller animal remains at the site were also described as charred, implying that they were burned by *H. floresiensis*, supporting the idea of a relatively small-brained yet behaviorally advanced fire-using hominin (*2*, *3*). Because hunting large game and controlling fire is generally associated with large-brained hominins, such as Neanderthals and modern humans (*7*–*9*), the association of these behaviors with *H. floresiensis* was, and continues to be, particularly unexpected. However, systematic taphonomic and zooarchaeological analyses of the Liang Bua *Stegodon* assemblage are needed to fully understand the extent of *H. floresiensis*’ dietary strategies and potential use of pyrotechnology.

We used taphonomic and zooarchaeological analyses (see Materials and Methods) to test whether *H. floresiensis* used fire and hunted *Stegodon* at Liang Bua. To determine the order of predator access to carcasses on a paleolandscape, comparative data such as mark locations and frequencies on prey long bones as well as prey element profiles are required (*10*, *11*). However, such data derive almost exclusively from the prey of mammalian predators and do not apply to the ecological context at Liang Bua, in which the only predator *H. floresiensis* would have competed with for access to *Stegodon* was *Varanus komodoensis* (Komodo dragon), the largest extant reptile on earth. Unlike mammals, Komodo dragons have serrated ziphodont dentitions that create a distinctive, complex patterning to the bone surfaces of their prey. We conducted a controlled feeding experiment at Zoo Atlanta to generate a sample of Komodo dragon tooth marks and compared these with experimentally generated cutmarks. We then randomly sampled 3155 *Stegodon* bone fragments (~27% of total assemblage) from stratigraphic Unit 1 [~190 to 60 thousand years ago (ka)] and Unit 2 (~60 to 50 ka), both of which are only associated with *H. floresiensis* (*12*, *13*), as well as 6906 murine rodent skeletal elements from stratigraphic Units 1 and 8, the latter of which is <11 ka and only associated with *H. sapiens* (Fig. 1) (see Materials and Methods). All 10,061 elements were examined for evidence of exposure to fire, and the *Stegodon* sample was further analyzed to identify the probable agent(s) responsible for the accumulation based on bone surface modifications and skeletal abundances.

## RESULTS

### Documenting skeletal damage in Komodo dragon prey

**Fig. 2.**
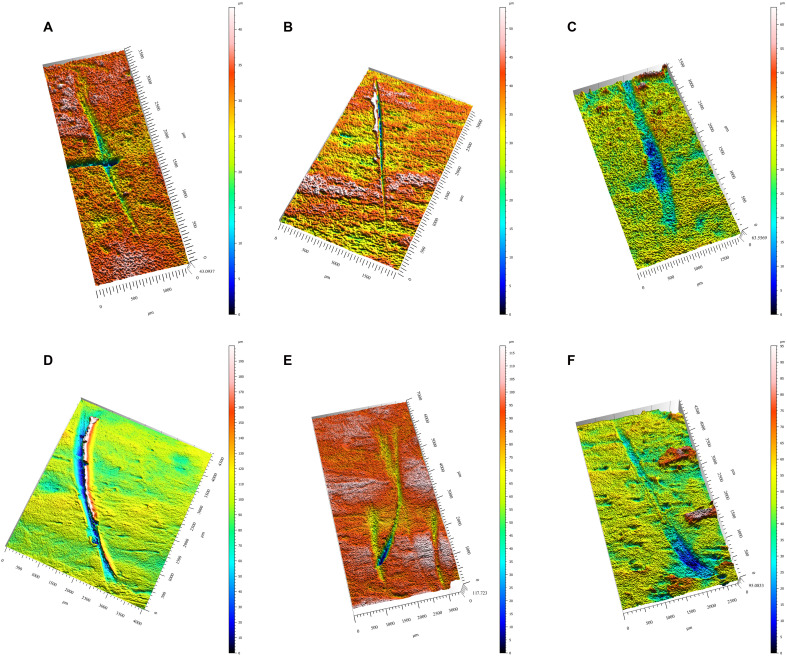
Models of tooth marks created by Komodo dragons. (**A** to **F**) Scores of Komodo dragon tooth marks generated from our experimental study.

To distinguish Komodo dragon tooth marks from cutmarks in the *Stegodon* assemblage, our controlled feeding experiment used a prepared adult goat carcass, with head and distal appendages removed, that was fed to a captive Komodo dragon. All remaining 72 bone elements were analyzed for tooth marks after feeding. Twenty-six of these elements had a total of 192 tooth marks with a mean of 7.4 marks per element (range: 1 to 55) with a majority found on the upper forelimb (42%, humeri; 14%, scapulae) (data S1). Scores made up ~95% of these marks, followed by pits (2.5%), notches, hooks, and furrows (<1% each). Twenty-seven scores contained internal microstriations, 2 had external microstriations, and 14 had asymmetrical microstriations emanating from the main groove to form a “fan” (Fig. 2, figs. S1 and S2, and table S1). These “fans” likely resulted when the posterior serrated edge of the tooth made contact with the bone during consumption. Score shape also varied depending on the type of bone and was likely the result of varied grip and rip actions (fig. S2 and movie S1). For example, scores found primarily on long bone shafts were typically shallower and wider compared to relatively deeper and narrower marks found on flatter and angular bones like ribs, vertebrae, and scapulae. In addition, depth was either consistent throughout the length of the score or varied in the form of a “pit and drag” effect. These results—in combination with those from a previous controlled feeding experiment involving Komodo dragons and multiple feeding sessions (*14*)—suggest that Komodo dragon tooth mark frequencies are highly variable but are consistently located on skeletal elements with “substantial” amounts of flesh.

**Fig. 3.**
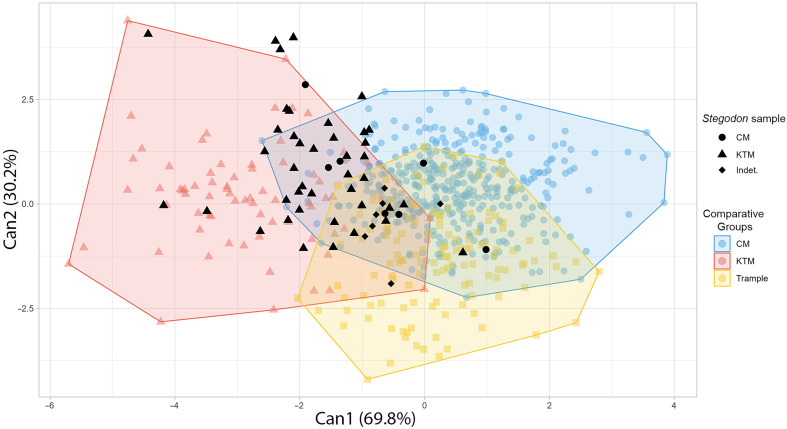
QDA of marks from the *Stegodon* assemblage and experimental samples. Analysis includes 3D variables plotted in canonical dimensions comparing trample marks (trample), cutmarks (CM), and Komodo dragon tooth marks (KTM) with marks from the Liang Bua *Stegodon* assemblage. Experimental samples are shown in yellow (trample), blue (cutmarks), and red (Komodo dragon tooth marks). The black icons represent identifications for the Liang Bua *Stegodon* sample. Liang Bua marks are labeled on the basis of high-confidence identifications, qualitative features, and posterior probability scores.

A three-dimensional (3D) quadratic discriminant analysis (QDA) using a resubstitution model classified the experimentally generated Komodo dragon tooth marks (*n* = 72), cutmarks (*n* = 403), and trample marks (*n* = 130) with 84% correct classification (Fig. 3 and table S2). The canonical scores between groups were statistically significant (*P* < 0.0001) (fig. S3). When plotted in canonical space, the Komodo dragon tooth marks separated from both cutmarks and trample marks along the negative end of the *x* axis (can1) (70%) with some overlap with both groups (Fig. 3). Trample marks and cutmarks clustered toward the positive end of can1 but with considerable overlap while separating primarily along the *y* axis (can2) (30%). Almost all the variables included in the analysis were statistically significant in separating cutmarks from Komodo dragon tooth marks (*P* < 0.05) apart from the maximum width (figs. S3 and S4). However, the width collected at the deepest point, as well as the depth, angle, and roughness, contributed the greatest for distinguishing Komodo dragon tooth marks from cutmarks and trample marks. Overall, Komodo dragon tooth marks tend to be shallower, shorter, and have a wider profile angle compared to cutmarks (fig. S5).

### Results from the *Stegodon* assemblage at Liang Bua

**Fig. 4.**
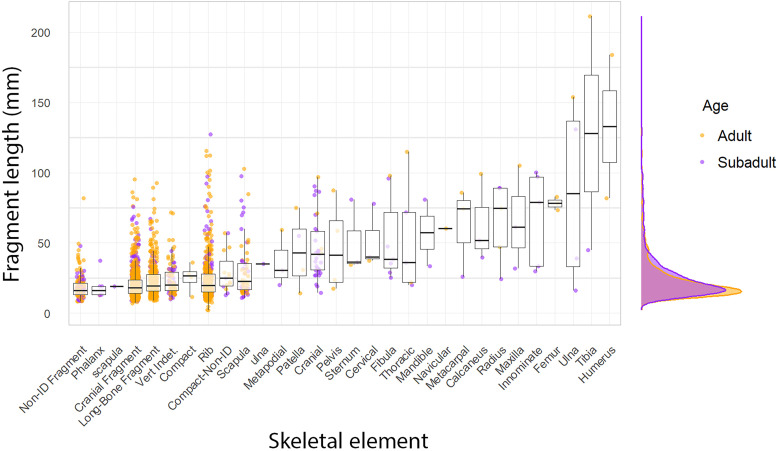
Size range and frequency of *Stegodon* skeletal elements. Boxplots of elements ordered by median values. Points are colored according to age-at-death (i.e., adult or subadult) with a density plot of each group displayed on the right. For the boxplots, center line is the median, upper and lower hinges are the first and third quartiles (25th and 75th percentiles), whiskers are 1.5 times the interquartile range, and points are all data points.

Among the *Stegodon* sample, 70% [709 subadult and 1511 adult bones and an additional 712 number of identified specimens (NISP)] were identified to elements but only 22% were sided to either left or right, enabling calculation of the minimum number of elements (MNE) (Fig. 4 and Table 1). On the basis of the most frequently represented skeletal elements, we calculated a minimum number of individuals (MNI) of nine subadults (unfused basiocciputs) and two adults (patellae), consistent with previously estimated demographic age profiles (*5*). Fragmentation rates (MNE/NISP) indicate that adult elements (0.06) were more fragmented compared to those of subadults (0.21). A large proportion of unidentifiable bone fragments was represented (29.8%), further demonstrating the high rate of fragmentation, most of which was due to postdepositional processes (60%) and recent breakage (28%) (table S3).

**Table 1.** Zooarchaeological summary of the *Stegodon* skeletal elements from Liang Bua used in this study.

Age (MNI)	Subadult (8.3)				Adult (1.9)				
Element	NISP	MNE	MAU	%RA	NISP	MNE	MAU	%RA	Total NISP
Cranial	29	10.30	8.30	62.05	4	2.00	1.00	52.63	33
Maxilla	2	0.90	0.90	5.42	1	0.50	0.50	13.16	3
Mandible	2	0.40	0.30	2.41	0	0.00	0.00	0.00	2
Stylohyoid	0	0.00	0.00	0.00	1	0.75	0.75	19.74	1
Clavicle	0	0.00	0.00	0.00	1	0.70	0.70	18.42	1
Sternum	2	1.80	1.80	21.69	1	0.70	0.70	18.42	3
Scapula	32	3.60	2.00	21.69	37	0.80	0.80	21.05	69
Cervical	2	0.70	0.10	1.20	1	0.50	0.07	13.16	3
Thoracic	3	1.40	0.07	0.84	2	1.60	0.08	42.11	5
Rib	123	7.90	0.29	2.38	387	6.60	0.99	173.68	510
Humerus	0	0.00	0.00	0.00	2	1.40	1.00	36.84	2
Radius	3	2.50	1.50	15.06	2	1.50	0.80	39.47	5
Ulna	4	2.00	1.00	12.05	1	1.00	1.00	26.32	5
Femur	0	0.00	0.00	0.00	4	1.50	1.50	39.47	4
Tibia	2	1.00	1.00	6.02	1	1.00	1.00	26.32	3
Fibula	6	2.00	1.00	12.05	1	1.00	1.00	26.32	7
Pelvic	5	2.80	1.80	16.87	6	1.00	1.00	26.32	11
Patella	1	0.80	0.80	4.82	3	1.90	1.90	50.00	4
Calcaneus	2	2.00	2.00	12.05	1	1.00	1.00	26.32	3
Compact	2	2.00	2.00	1.00	2	2.00	2.00	52.63	4
Metapodial	3	3.00	0.15	1.81	3	2.70	1.85	71.05	6
Phalanx	5	4.00	0.18	2.19	1	1.00	1.00	26.32	6
Compact Indet.	3				13				16
Vert Indet.	40				56				96
Long-bone fragment	69				338				407
Cranial fragment	369				642				1011
Non-ID fragment	52				182				234

A total of 243 marks were identified as possible predatory damage on 62 *Stegodon* bones (5% of the sampled assemblage). A sample (*n* = 55) of these marks were molded from durable bones and surface scanned to generate 3D topographic models (see Materials and Methods; data S2). These models were then analyzed using the QDA model of experimentally generated Komodo dragon tooth marks, cutmarks, and trample marks (Fig. 3 and data S3). Results with high classification accuracy (85%) showed that, as in the experimental results, Komodo dragon tooth scores tend to be shallower and shorter compared to cutmarks, with a greater profile angle (i.e., wider) and maximum width (figs. S3 to S5). Fifty-one additional marks were analyzed using 2D methods with moderately high classification accuracy (74%) (figs. S3 and S6, table S4, and data S4 and S5). The remaining 137 marks were evaluated on the basis of qualitative features and labeled as either high-confidence cutmarks or Komodo dragon tooth marks.

**Fig. 5.**
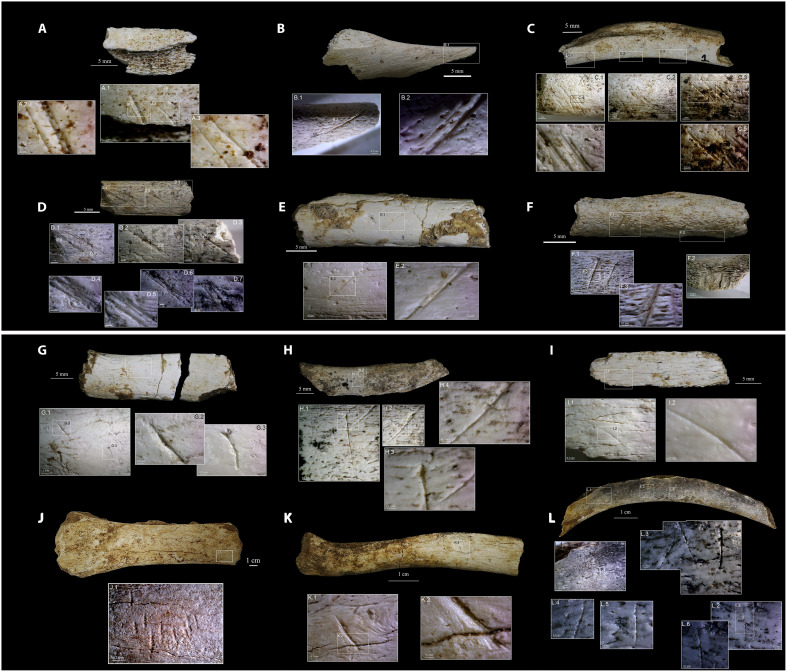
Selected bone fragments showing marks made by *H. floresiensis* and Komodo dragons. (**A**) Rib fragment (ID 132) with cutmarks highlighted in A.1 and A.2. (**B**) Rib fragment (ID 574) with one cutmark highlighted in B.1 and B.2. (**C**) Rib fragment (ID 282) with cutmarks highlighted in C.1 to C.5. (**D**) Rib fragment (ID 247) with cutmarks shown in D.1 to D.7. (**E**) Rib fragment (ID 637) with a cutmark shown in E.1 and E.2. (**F**) Weathered rib fragment (ID 491) with cutmarks highlighted in F.1 and F.3. Rodent gnawing is shown in F.2. (**G**) Sternal fragment (ID 643) with two tooth marks highlighted in G.1 to G.3. (**H**) Rib fragment (ID 527) with tooth marks shown in H.1 to H.4. (**I**) Long bone fragment (ID 627) with one tooth mark highlighted in I.1 and I.2 with curvature typical of varanid tooth scoring (*15*). (**J**) Distal femoral diaphysis (ID 230) with clustered tooth marks shown in J.1. (**K**) Partial first rib (ID 340) with one tooth mark shown in K.1 with a microstriation “fan.” (**L**) Burned rib fragment (ID 353) with clustering of scores shown in L.1 to L.6.

Overall, 154 marks on *Stegodon* bones indicate predatory damage by either hominins (i.e., cutmarks) or Komodo dragons (i.e., tooth marks) (Fig. 5 and table S5). Fifty-four marks from 20 bones were identified as cutmarks based on macroscopic observations of features and results from the QDA. Cutmarks occurred on the dorsal aspect of an intermediate phalanx (3 marks), a radius (1 mark), nine rib fragments (27 marks), a thoracic vertebra (1 mark), a cranial fragment (1 mark), a hyoid bone (3 marks), one innominate (1 mark), and five long bone fragments (17 marks). Of these bones, 13 were adult (41 marks) and 7 were subadult (13 marks), indicating that *H. floresiensis* butchered both adult and juvenile *Stegodon* carcasses at the site. Neither percussion marks nor projectile damage were identified in the assemblage.

One hundred marks from 31 bones were identified as Komodo dragon tooth marks based on identifiable features and results from the QDA (Figs. 3 and 5). These tooth marks were identified on two cranial fragments (2 marks), the distal end of a fibula (7 marks), a femoral shaft (15 marks), a metapodial (1 mark), an ulnar shaft (2 marks), a radial shaft (3 marks), 23 rib fragments (52 marks), five long bone fragments (10 marks), a sternal fragment (2 marks), and an unidentified bone (6 marks). Of these, 30 bones were adult (79 marks) and 7 were subadult (21 marks). Clustering of two or more marks were identified, 42% of which were in parallel clusters, 12% in nonparallel clusters, and 45% were isolated. For curvature estimates, 78% of marks measured straight (0°), 19% measured <45°, and 2% measured between 45° and 90°. Six marks retained a microstriation “fan.”

**Fig. 6.**
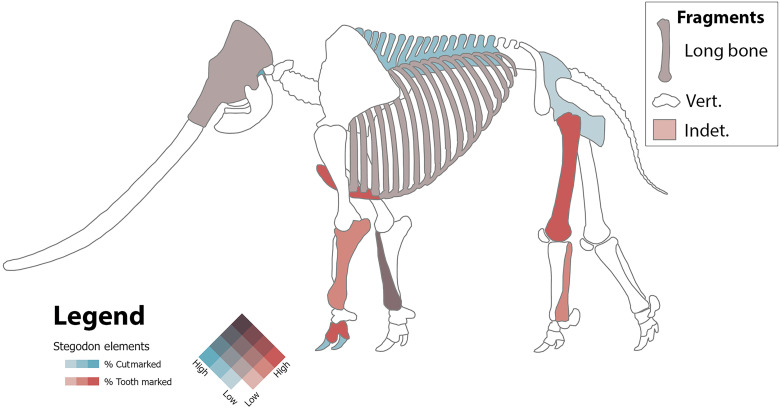
Distribution of marks by skeletal elements identified in the *Stegodon* assemblage. Colors indicate the frequencies of marks relative to element abundance. Image generated using ArcGIS Pro.

Overall, predatory damage in the sampled assemblage is low (5.7% adult and 0.82% subadult) with a high proportion of cutmarks and Komodo dragon tooth marks identified on adult (76 and 79%, respectively) compared to juvenile bones (24 and 21%, respectively). Although many rib elements showed predatory damage, this is likely due in part to recognition bias because ribs were frequently more recognizable in this fragmentary assemblage compared to other elements (Table 1). When element frequency is controlled for, the highest relative frequencies of cut-marked skeletal elements were the stylohyoid (100%), radius (20%; i.e., one cut-marked radius of five radii in total), thoracic vertebra (20%), phalanx (16.7%), cranial bones (3%), ribs (1.8%), and long bone fragments (0.8%). Conversely, the highest relative frequencies of Komodo dragon tooth-marked bones were the metapodial and sternum (33.3%), femur (25%), radius and ulna (20%), fibula (14.3%), rib (4.5%), long bone fragments (1.3%), and non-ID fragments and cranial fragments (<1%) (Fig. 6).

### Evidence of fire use

**Fig. 7.**
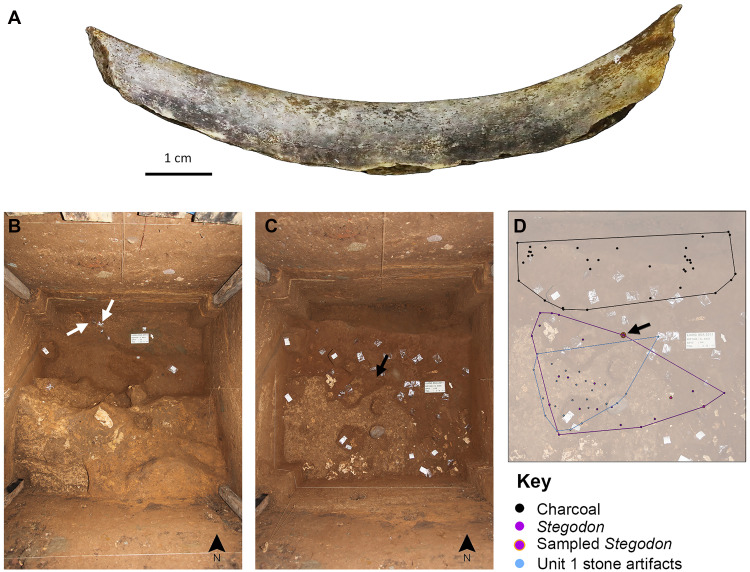
*Stegodon* rib (LB-STG-353) and its recovery location near a stratigraphic unconformity in Sector XXII. (**A**) Photograph of LB-STG-353 showing the evidence of burning damage. (**B**) Spit 43 in the northern half of Sector XXII containing two charcoal fragments dated to ~10,910 and ~10,940 BP (before the present) (white arrows). The yellowish-brown colored sediments in the southern half of the square are *H. floresiensis* and *Stegodon*-bearing deposits (>60 ka), whereas the reddish-brown sediments in the northern half are unconformably overlying deposits (<20 ka). (**C**) Spit 44 showing the removal of layers in the southern half of the square. (**D**) Overlay of 3D-plotted charcoal, *Stegodon* (including bones sampled in this study), and stone artifacts recovered from spit 44 with convex hulls showing the distributional extent of each group. In (C) and (D), a black arrow shows the recovery location of specimen LB-STG-353.

Only 1 of the 3155 *Stegodon* elements (0.0003%)—a rib fragment recovered from Sector XXII at 4.45-m depth—showed signs of nonlocalized burning damage (*15*, *16*) (Fig. 7). This particular fragment was found situated along an east-west unconformity where older in situ sediments (>60 ka) associated with *H. floresiensis* accumulated in the southern half of the square and much younger sediments (<20 ka) associated with *H. sapiens* accumulated in the northern half (*17*). The juxtaposition of this burnt bone with the unconformably overlying sediments suggests that this bone was likely exposed close to the cave floor surface long after burial when *H. sapiens* constructed hearths and hearth-like structures in the cave (*18*).

**Fig. 8.**
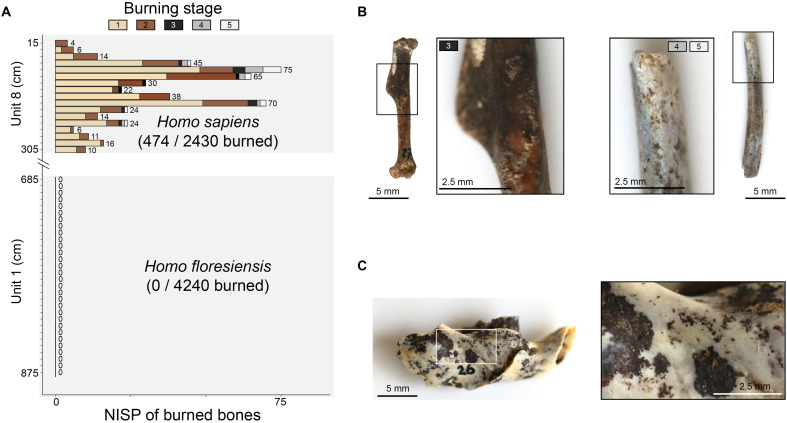
Evidence of burning damage from murine skeletal remains. (**A**) Data from Veatch (*19*) showing the number (NISP) of burned bones according to spit from Sector XI. Units associated with *H. sapiens* (Unit 8; <~11 ka) and *H. floresiensis* (Unit 1; ~190 to 60 ka) are delineated. (**B**) Photographs of a carbonized murine humerus and calcined rib from Unit 8 compared to (**C**) a manganese-stained mandible from Unit 1. Notice the dark dendritic and spotty patterning of manganese compared to the more homogeneous coloration of the carbonized bone.

Murines are notably abundant and consistently represented at Liang Bua providing the best comparison of burning damage between stratigraphic units associated with either *H. sapiens* or *H. floresiensis* (*13*). Of 2430 bone and dental elements from Unit 8, associated with *H. sapiens*, 474 elements (~20%) showed evidence of burning, ranging from spotting (stage 1) to calcined (stage 5) (Fig. 8). Conversely, 0% of 4240 murine elements sampled from Unit 1, associated with *H. floresiensis*, showed any traces of burning damage. Instead, bones from Unit 1 were often stained by manganese (*19*), which is common throughout the Liang Bua stratigraphy and typically results in distinctive black dendritic and spotting patterns on bone surfaces due to the presence of manganese oxide in the sediments (*18*) (Fig. 8).

## DISCUSSION

The controlled use of fire and coordinated hunting of large game are considered critical milestones in hominin evolution because they are the result of high-fidelity social learning through cumulative culture and cooperation (*20*). The fact that *H. floresiensis* was originally described as having these behavioral adaptations continues to be a source of intense debate. However, with no evidence of in situ burning in the *Stegodon* or murine assemblage sampled thus far from *H. floresiensis*–bearing sediments at Liang Bua, it is reasonable to conclude that past fire use at the site was the sole result of *H. sapiens* behavior that occurred from ~46 ka until present day, well after *H. floresiensis* and *Stegodon* disappeared from the area (*13*, *17*, *18*, *21*). Moreover, the presence of cut-marked *Stegodon* bones at the site suggests that *H. floresiensis* accessed some of these proboscidean carcasses for dietary purposes and thus would have consumed this valuable resource raw. Although the earliest widely accepted evidence of hominin fire use occurs ~1 million years ago (Ma) in Acheulean deposits at Wonderwerk Cave, South Africa (*22*), evidence for habitual fire use does not appear until 420 ka at Qesem Cave, Israel (*7*). Thus, it is perhaps not unexpected that fire use was apparently not part of the technological repertoire of *H. floresiensis* or even its direct ancestors that first reached Flores likely before 1 Ma (*23*–*25*).

Although the taphonomic evidence indicates that *H. floresiensis* accessed proboscidean carcasses for food, whether these hominins acquired this dietary resource through coordinated hunting or some form of scavenging (e.g., opportunistic, competitive, etc.) is an important question. However, distinguishing among these behaviors in the archaeological record is challenging and frequently contested (*26*–*29*) as is determining the order in which other predators accessed prey (Table 2). Successful hunting by *H. floresiensis* would have provided primary access, but so too would being the first predator on site following a natural death or perhaps one spurred on by a Komodo dragon bite.

**Table 2.** Hunting and access scenarios for Komodo dragons and *H. floresiensis* at Liang Bua with supporting evidence specifying primary versus secondary access for each predator.

Scenario	Komodo dragons hunt–Hominins scavenge			Hominins hunt–Komodo dragons scavenge		
	Order of access	Evidence needed to support	Evidence confirmed	Order of access	Evidence needed to support	Evidence Confirmed
	Komodo-Hominin: Komodo dragons arrive at the location of their prey (Liang Bua) before hominins access the remains.	Removal of elements retaining tissue after Komodo dragons’ feed	Yes	Hominin-Komodo: Hominins get primary access from hunting followed by Komodo dragons who track the decomposing prey using their sense of smell.	Projectile technology and impact damage	No
		Cutmarks on low-utility elements	Yes		Greater frequency of cutmarks on high-utility elements	No
		Komodo dragon tooth marks on high-utility elements	Yes		Greater frequency of Komodo dragon tooth marks on low-utility elements	No
					High-utility elements present at Liang Bua via transport from the kill location	No
	Hominin-Komodo: After *Stegodon* is wounded from a Komodo dragon bite, hominins access the deceased *Stegodon* before Komodo dragons arrive at the death site (Liang Bua) using their sense of smell.	High-utility skeletal elements were removed to consume elsewhere	Possibly*	Hominin only: Hominins kill and consume prey without scavenging from Komodo dragons.	Projectile technology and impact damage	No
		Greater frequency of cutmarks on high-utility elements (those not removed)	No		Only cutmarks on skeletal elements	No
		Greater frequency of Komodo dragon tooth marks on low-utility elements	No		Only have high-utility elements present at Liang Bua via transport from the kill location	No
	Komodo only: Komodo dragons kill, track (to Liang Bua), and consume prey without scavenging from hominins.	Komodo dragon tooth marks only	No			
		Relatively even distribution of skeletal elements (minus smaller elements that were deleted via ingestion by Komodo dragons)	Possibly*			

* There is a low number of high-utility elements in the sampled *Stegodon* assemblage, but they are not absent.

On the basis of the behavior of extant elephants, ancient proboscideans may have been attracted to caves as places to mourn deceased individuals (*30*, *31*) and to seek relief from heat and/or for sources of water, salt, and minerals (*32*). For example, observations of both African and Asian elephants include digging for water and minerals such as manganese, which is present in the sediments of Liang Bua, and may have been an attractive feature for *Stegodon* (*18*). The MNE values for adult *Stegodon* are relatively even, suggesting that these individuals likely died on site. Conversely, the MNE values for juveniles are dominated by cranial elements, which modern foragers are known to transport only over short distances due to their weight and relatively low utility (*33*). High-utility elements like the femur and humerus are absent (or unidentifiable due to high fragmentation rates) in the juvenile skeletal material, whereas distal limb elements that are more likely to be available to scavengers are present and cutmarked (*11*). One possible explanation for the slight unevenness of skeletal elements between adults and juveniles is that either some high-utility juvenile elements were consumed entirely by Komodo dragons [i.e., swallowed whole (*34*)] or were removed from the site by *H. floresiensis* to consume elsewhere if and when they may have had early access to a *Stegodon* carcass.

### Komodo dragon feeding ecology

Komodo dragons are extremely skilled predators, ambushing unsuspecting prey while also relying on their keen sense of smell to locate decomposing flesh from up to several kilometers away (*34*). Once bitten by a Komodo dragon, a concoction of toxic venom is released into the prey’s bloodstream causing systemic infection preventing coagulation and the prey is often consumed on site (*35*). In other cases, prey will escape and die within a week after being injected with venom, after which Komodo dragons will consume the carcass at the location where the prey died; these reptilian predators are not known to relocate carcasses for later consumption (*34*). Our experimental study as well as that by D’Amore and Blumenschine (*14*) showed that Komodo dragons do not crush or chew on bones—a behavior that is often observed in other carnivores, such as hyenas and lions (*36*, *37*). Instead, Komodo dragons use their curved and serrated teeth to essentially tear and consume as much soft tissue as possible using the “grip-and-rip” method where the animal latches its jaw onto the prey, locks its mandible, and moves its head side-to-side to loosen the tissue (*38*) (movie S1), sometimes leaving as little as 12% of the carcass (depending on the relative size of the carcass) if not consumed whole (*39*). Although this feeding behavior has been observed to be quite consistent (*14*), the outcome can vary extensively, leaving zero to ~330 traces or more of tooth damage from a single individual. Because Komodo dragons can also be communal feeders (*34*), the frequency of tooth marks may vary depending on the ecological circumstances of the encounter.

The pattern of Komodo dragon tooth marks observed in the *Stegodon* assemblage at Liang Bua is consistent with tooth mark distribution when these giant varanids have primary access to carcasses (Fig. 6 and table S1) (*14*). For example, Komodo dragon tooth scores were concentrated largely on elements yielding greater meat volumes that are targeted during consumption, such as the forequarter and hindquarter (data S1). In contrast, cutmarks were found on distal appendages (i.e., phalanx), cranial bones, thoracic vertebrae, and phalanges, which is not an expected pattern if hominins had primary access to such large-bodied prey either through scavenging or hunting. When scavenging, *H. floresiensis* would not have been at risk after consuming meat from Komodo dragon kills. Komodo dragon venom consists of proteins that are too large to pass through human stomach linings and would be broken down by stomach enzymes (*35*).

### Human behavioral ecology of hunting proboscideans

The most convincing evidence for hunted proboscideans by hominins consists of projectile damage to the bone, either by embedded tools or impact damage (*40*). At Liang Bua, there is no evidence that *H. floresiensis* used projectile technology, so capturing these relatively large animals would have been extremely risky. Modern analogs of humans having primary access to proboscidean carcasses are currently based on cutmark distributions on limb bones and ribs and are further limited in application to the archaeological record due to several unknown factors such as the condition of the remains and butcher objectives (*41*, *42*). Moreover, butchering elephant bones leaves fewer marks compared to other large-bodied prey due to the relatively greater tissue mass and thicker periosteum (*41*) and may partly explain the low cutmark frequencies observed on the Liang Bua *Stegodon*.

The ethnographic record shows that hunting elephants results in higher handling costs and lower postencounter return rates compared to hunting other large game such as zebra or buffalo (*43*). Elephants are among the lowest ranked prey species when considering economic measures of success rates, edible weight, as well as pursuit, handling, and butchering times (*43*). If Liang Bua *Stegodon* (or its larger ancestor, *Stegodon florensis florensis*) and giant rat (*Papagomys armandvillei*) are included for comparison, the giant rat ranks higher (#9) compared to adult and subadult *Stegodon* (#17, #19, and #20) (table S6). This suggests that, although *H. floresiensis* would gain a large total caloric return by successfully hunting *Stegodon*, the costs involved could potentially outweigh any social and/or caloric advantages. Moreover, the smell of dead, decomposing *Stegodon* at Liang Bua would have likely attracted Komodo dragons from within several kilometers, creating a high-risk scenario for *H. floresiensis*.

Establishing clear causal links between human or other accumulating agents and faunal assemblages, rather than relying solely on depositional associations, is a critical component of modern zooarchaeological theory and practice (*44*). Initial interpretations of *H. floresiensis* behavior were based almost entirely on depositional association, and thus our results provide a more critical and nuanced interpretation of ancient hominin dietary and technological strategies in a cave environment in Island Southeast Asia. With no indications of carcass transport to Liang Bua, the cave was clearly a central location that attracted *Stegodon* between ~190 and 50 ka. The overall taphonomic evidence suggests that the *Stegodon* assemblage reflects a combination of mostly primary access by Komodo dragons and secondary access by *H. floresiensis* where both predators consumed *Stegodon* ranging from neonates to adults, as well as natural *Stegodon* deaths. The lack of any single, individual bone that retains both cutmarks and Komodo dragon tooth marks suggests that these two predators were probably not consuming resources from the same skeletal parts of any one *Stegodon* carcass. However, feeding trace models of hominin and mammalian carnivore carcass consumption have demonstrated that, in some cases, specimens bearing both butchery marks and carnivore tooth marks are not produced after access by both predators (*28*). Regardless, *H. floresiensis* clearly interacted with these prey animals, but there is no evidence yet to support the hypothesis that *H. floresiensis* actively hunted *Stegodon*. Instead, the observed pattern of Komodo dragon tooth marks concentrated on high-utility *Stegodon* bones at Liang Bua coupled with low frequencies of hominin cutmarks mainly on low-utility elements and no projectile impact damage suggest that *H. floresiensis* likely engaged in some form of passive scavenging and consumed *Stegodon* meat raw. In addition, the unevenness of the juvenile skeletal part profiles suggests that some high-utility elements, like the humerus and femur, were selectively removed and consumed elsewhere.

*H. floresiensis* was initially described as “capable of complex behavior and cognition” (*2*) in part because this species may represent an isolated island-dwarfed descendant lineage of *H. erectus* sensu stricto (*1*, *2*). However, evidence for behavioral complexity in *H. floresiensis*, including complex tool and fire use, have weakened considerably over time (*18*, *21*, *45*–*47*). Without fire, *H. floresiensis* would not likely have evolved adaptations in gut physiology and masticatory anatomy that maximize energy acquisition from consuming cooked foods, as seen in some other hominins (*8*, *48*). Furthermore, *H. floresiensis* retains postcranial anatomy as well as relative arm, leg, and foot proportions unconducive for running and throwing that would make the act of hunting large game (in the traditional sense) quite difficult (*49*–*56*). The evidence to date thus suggests that *H. floresiensis* did not engage in a behavioral repertoire as diverse or as flexible as in modern humans or Neanderthals, possibly due to an ancestry in which large game hunting and controlled use of fire did not evolve (*1*, *2*, *57*).

## MATERIALS AND METHODS

Our analyses included the documentation of linear bone surface modifications indicative of predation by hominins and varanids from a sample of the *Stegodon* bone assemblage from Liang Bua. We determined the effector using noncontact profilometry methods (*58*) and zooarchaeological approaches to determine the predatory order of access. In addition, burning patterns were analyzed from a sample of rodent bones from stratigraphic units associated only with *H. floresiensis* and compared with those associated with *H. sapiens*.

### Sample and stratigraphic context

The *Stegodon* assemblage at Liang Bua derives from stratigraphic Units 1 and 2, which occur immediately beneath Tephra 1 (~60 ka) and Tephra 3 (~50 ka), respectively (Fig. 1) (*12*, *13*). These units are dated between ~190 and 50 ka, contain no anatomical or behavioral evidence of *H. sapiens*, and are instead associated only with *H. floresiensis* (*12*, *13*). Although some *Stegodon* remains have also been recovered from Units 4, 5, 6, and 8A, previous research has shown that these elements do not represent animals that died during the depositional accumulation of these units (*12*). Instead, these elements have been reworked from older pedestal deposits of Units 1 and 2 that occur vertically higher than younger, unconformably overlying sediments that were deposited in front (downslope) of the remnant pedestal (*12*).

Our study sample consisted of nondental skeletal remains of *Stegodon* from Liang Bua, which we randomly sampled from Sectors I, III, IV, VII, XI, XIV, XV, XVII, XXI, XXII, and XXIII. Because all the sampled *Stegodon* bones are associated only with *H. floresiensis*, results for skeletal part profiles, mark identifications, and mark frequencies are reported as one assemblage (Table 1). Jitter plots in fig. S7 show the recovery locations (sectors, depth, and stratigraphic unit) of the entire study sample by mark type (cutmark, tooth mark, and no mark).

In situ murine rodent remains were sampled from stratigraphic Units 1 and 8 and only from Sector XI to identify the presence of fire use in units associated with *H. floresiensis* and *H. sapiens*, respectively. Unit 8 was selected for comparison with Unit 1 because it is well dated (~11 ka to present), contains the least amount of eroded and/or reworked sediments from the preceding units, and contains unambiguous skeletal and behavioral evidence of *H. sapiens* (*12*).

Sectors I, III, and IV represent 3 m–by–3 m areas excavated during field seasons that took place between 2001 and 2003 (*59*). Each of the other sectors represents a 2 m–by–2 m area excavated during field seasons that took place between 2003 and 2011 (*17*, *59*). Sectors were excavated in 10-cm intervals (referred to as spits) while following geological layers. Findings that were visible upon excavation were plotted in 3D space. Sediments in each spit were hand sieved followed by wet sieving with a 2-mm mesh. Recovered findings were cleaned, sorted, cataloged in bags, and transported to Pusat Penelitian Arkeologi Nasional (now Badan Riset dan Inovasi Nasional in Jakarta, Indonesia) for curation and further study.

### Taphonomic and zooarchaeological analyses

*Stegodon* bones larger than 10 mm were included in the analysis, including some small fragments that were broken due to long-term curation damage from larger bones. Bone surface visibility was graded on a 10% scale based on the amount of visible cortical bone surface. Bone color and fossilization level were qualitatively described and grouped. Long bone breakage (*60*) and fragmentation (NISP/MNE) were recorded. Where possible, skeletal element portions, side (left or right), and age (adult or juvenile based on the presence or absence of lamellar or woven bone, respectively, as well as epiphyseal and suture fusion) of each specimen were recorded. Standard zooarchaeological indices were recorded as summarized by Lyman in table 1 of (*61*) and include the following:

Number of individual specimens (NISP): “number of identifiable specimens” (Payne, 1975).

Minimum number of elements (MNE): “minimum number of skeletal elements; determined from the most common portion of each skeletal element [recorded on a 10% scale] and summing right and left sides [for paired elements]” (Stiner 1991).

Minimum anatomical unit (MAU): “minimum animal units” (Binford 1984) calculated as

$${\text{MNE}}_{e}/\text{number of times}\;e\;\text{occurs in}\;\text{one}\;\text{complete skeleton}$$

Minimum number of individuals (MNI): “the minimum number of individual animals represented by each anatomical part” (Binford and Bertram 1977).

Percent relative abundance (%RA): “the relative abundance of element i” (Andrews 1990; Dodson and Wexlar 1979; Brain 1969) calculated as

$$100*\left[{\text{MNE}}_{i}/\left[\text{MNI}*E_{i}\right]\right]$$

Linear bone surface modifications were identified using a 10X hand lens and a Dinolite Edge series microscope (20X–220X) and preliminarily assigned to an actor based on established criteria on a high to low confidence scale (*62*–*65*). Silicone molds were only able to be collected from a sample of marks from nonfragile *Stegodon* bones (i.e., bones that could not break when the mold was taken) and quantitatively analyzed using comparative datasets (see below). Visualization for the locations of marks on the *Stegodon* assemblage were done using ArcGIS Pro (3.2.0) on templates created by the authors. Whereas mark percentages tend to be reported on the basis of long bong counts (and on the basis of bovid assemblages), cutmark and Komodo dragon tooth marks are reported here on the basis of the percentage of the entire assemblage. This was done because the *Stegodon* bone assemblage is highly fragmentary (most bones measuring <50 mm in length) and long bone fragments were relatively scarce. Weathering stages (*66*), burning (*16*, *67*), postdepositional damage (*68*, *69*), and manganese oxide staining (*67*) were used as defined in the literature. Broken edges for all bone fragments were compared to the bone surface to determine whether breaks were in situ, postdepositional, or recent break.

### Experimental comparative samples

A collection of marks of known origin was used as comparative datasets for statistical analysis. These included a sample of experimental butchery marks from medium and large mammal long bone diaphyses (*70*) and experimentally generated trample marks from cows stepping on bones. To obtain a sample of Komodo dragon tooth marks, we conducted a small, controlled feeding experiment [this research was reviewed and approved by the Zoo Atlanta Scientific Research Committee (approval no. SRC 2016-20)]. One headless dressed goat carcass was fed to a single Komodo dragon at Zoo Atlanta. The goat was harnessed to a log inside the enclosure to ensure the Komodo dragon would not drag the carcass out of sight of the observers. The feeding was video recorded and general behavior was noted (movie S1). The remaining bones were collected and cleaned using 1 part hydrogen peroxide (3% solution) and 2 parts water and analyzed for bone surface modifications. Tooth scores and pits were identified using a 10X hand lens and a Dinolite Edge series microscope (20X–220X) (figs. S1 and S2). The location and qualitative features of each mark were recorded as well as any other bone damage caused by the Komodo dragon during feeding.

### Statistical analysis

Marks were quantified following the protocol established by Pante *et al.* (*58*). Molds of marks from the *Stegodon* assemblage (*n* = 55) and the comparative datasets (Komodo dragon tooth marks = 72; cutmarks = 403; trample = 130) were scanned using a Sensofar S Neox 3D optical noncontact profilometer to generate 3D topographic profiles and models of each mark. The resolution of the *x* and *y* axes is 2.76 μm, whereas the *z*-axis resolution is 70 nm. Data processing and analysis were performed using Digital Surf’s Mountains and included removing outliers, filling missing data points, and removing the underlying form of the bone (*58*). 3D variables include the surface area, volume, maximum depth, mean depth, maximum length, and maximum width. Additional variables were measured from a profile taken at the maximum depth of a mark including area of the hole, depth of a transverse profile, roughness (Ra), angle, and radius of the hole. All data were normalized using the box-cox method in PAST. A QDA was performed in RStudio (2023.06.0+421) using the MASS and CANDISC packages (*71*–*73*). Both resubstitution and cross-validation models were performed, and we chose to use the resubstitution model because the differences between the two were minor. The resubstitution model accuracy for equal prior probabilities is 85 and 82% when analyzed for cross-validation (see the Supplementary Text for QDA model). The resubstitution model identified Komodo dragon tooth marks with greater accuracy (95.8%) than cutmarks (85.6%) and trample marks (79.2%). This is likely due to greater similarity between cutmarks and trample marks (see below).

An additional 2D analysis using ImageJ was performed to distinguish Komodo dragon tooth marks from cutmarks from marks on bones that were too fragile to mold. Photographs of marks from the *Stegodon* assemblage (*n* = 51) were compared with Komodo dragon tooth marks (*n* = 64) and cutmarks (*n* = 64) of known origin. The cutmark sample in this analysis comes from a collection of three experimentally butchered white-tailed deer (*Odocoileus virginianus*) bones using experimentally created Oldowan replica stone tools from basalt, chert, and obsidian. Using ImageJ, the maximum length, maximum width, area, angle, perimeter, roundness, circularity, and aspect ratio were collected from photographs of each mark. As described above, all data were normalized using the box-cox method in PAST and a QDA using the resubstitution model was performed in RStudio with 74% correct classification.

All marks were then evaluated on the basis of the 3D and 2D analysis and qualitative features, and a final mark assessment [Komodo tooth mark (KTM), cutmark (CM), or Indet.] was made. If applicable, a high-confidence ID based on qualitative features was assigned to additional marks that could not be included in either the 3D or 2D analysis.

A list of variables used in the 3D analysis is defined below (note the difference in angle).

1) Surface area: area in μm^2^ of the defined plane (i.e., outline of the mark).

2) Volume: volume in μm^3^ of the bone displaced by the mark.

3) Maximum depth: the greatest length from the top of the defined plane to the bone within the defined area.

4) Mean depth: the arithmetic means of depth within the defined area.

5) Maximum length: longest straight distance of the plane.

6) Maximum width: longest distance perpendicular to the longest axis of the plane.

7) Area of the hole: the area of a profile plane (μm^2^) made at the maximum depth of the mark.

8) Depth of a transverse profile: the depth of the profile plane.

9) Roughness (Ra): the arithmetic mean deviation from the roughness profile.

10) Angle: the angle between two segments of the profile drawn between the deepest point and the first and last measured points (i.e., the top of the marks’ walls).

11) Radius of the hole: an arc drawn between the first and last points of the profile and measures the best fit for all the points in the profile.

A list of variables used in the 2D analysis is defined below (note the difference in angle).

1) Major (maximum length): primary axis of the best fitting ellipse (i.e., polygon).

2) Minor (maximum width): secondary axis of the best fitting ellipse.

3) Area: area of selection in calibrated units (mm^2^).

4) Angle: the angle of the mark midline relative to the axis of the bone.

5) Perimeter: the length of the outside boundary of the selection.

6) Roundness: 4 * area/(π * major_axis^2^), or the inverse of the aspect ratio.

7) Circularity: 4π * area/perimeter^2^. A value of 1.0 indicates a perfect circle. As the value approaches 0.0, it indicates an increasingly elongated shape.

8) Aspect ratio: the ratio between the maximum length and width.

## Supplementary Materials

### The PDF file includes:

Supplementary Text Figs. S1 to S7 Tables S1 to S6 Legends for data S1 to S5 Legend for movie S1 References

## References

[1] P. Brown, T. Sutikna, M. J. Morwood, R. P. Soejono, Jatmiko, E. W. Saptomo, R. A. Due, A new small-bodied hominin from the Late Pleistocene of Flores, Indonesia. Nature 431, 1055–1061 (2004). 15514638 10.1038/nature02999

[2] M. J. Morwood, P. Brown, Jatmiko, T. Sutikna, E. W. Saptomo, K. E. Westaway, R. A. Due, R. G. Roberts, T. Maeda, S. Wasisto, T. Djubiantono, Further evidence for small-bodied hominins from the Late Pleistocene of Flores, Indonesia. Nature 437, 1012–1017 (2005). 16229067 10.1038/nature04022

[3] M. J. Morwood, R. P. Soejono, R. G. Roberts, T. Sutikna, C. S. M. Turney, K. E. Westaway, W. J. Rink, J.-x. Zhao, G. D. van den Bergh, R. A. Due, D. R. Hobbs, M. W. Moore, M. I. Bird, L. K. Fifield, Archaeology and age of a new hominin from Flores in eastern Indonesia. Nature 431, 1087–1091 (2004). 15510146 10.1038/nature02956

[4] G. D. van den Bergh, H. J. M. Meijer, R. A. Due, M. J. Morwood, K. Szabó, L. W. van den Hoek Ostende, T. Sutikna, E. W. Saptomo, P. J. Piper, K. M. Dobney, The Liang Bua faunal remains: A 95 k.yr. sequence from Flores, East Indonesia. J. Hum. Evol. 57, 527–537 (2009). 19058833 10.1016/j.jhevol.2008.08.015

[5] G. D. van den Bergh, R. D. Awe, M. J. Morwood, T. Sutikna, Jatmiko, E. W. Saptomo, The youngest *Stegodon* remains in Southeast Asia from the Late Pleistocene archaeological site Liang Bua, Flores, Indonesia. Quat. Int. 182, 16–48 (2008).

[6] D. Falk, C. Hildebolt, K. Smith, M. Morwood, T. Sutikna, P. Brown, Jatmiko, E. W. Saptomo, B. Brunsden, F. Prior, The brain of LB1, *Homo floresiensis*. Science 308, 242–245 (2005). 15749690 10.1126/science.1109727

[7] R. Barkai, J. Rosell, R. Blasco, A. Gopher, Fire for a reason: Barbecue at Middle Pleistocene Qesem Cave, Israel. Curr. Anthropol. 58, S314–S328 (2017).

[8] R. Wrangham, Control of fire in the paleolithic: Evaluating the cooking hypothesis. Curr. Anthropol. 58, S303–S313 (2017).

[9] K. Isler, C. P. van Schaik, How humans evolved large brains: Comparative evidence. Evol. Anthropol. 23, 65–75 (2014). 24753347 10.1002/evan.21403

[10] R. J. Blumenschine, Carcass consumption sequences and the archaeological distinction of scavenging and hunting. J. Hum. Evol. 15, 639–659 (1986).

[11] R. J. Blumenschine, K. A. Prassack, C. D. Kreger, M. C. Pante, Carnivore tooth-marks, microbial bioerosion, and the invalidation of Domınguez-Rodrigo and Barba’s (2006) test of Oldowan hominin scavenging behavior. J. Hum. Evol. 53, 420–426 (2007). 17727916 10.1016/j.jhevol.2007.01.011

[12] T. Sutikna, M. W. Tocheri, M. J. Morwood, E. W. Saptomo, Jatmiko, R. A. Due, S. Wasisto, K. E. Westaway, M. Aubert, B. Li, J.-x. Zhao, M. Storey, B. V. Alloway, M. W. Morley, H. J. M. Meijer, G. D. van den Bergh, R. Grün, A. Dosseto, A. Brumm, W. L. Jungers, R. G. Roberts, Revised stratigraphy and chronology for *Homo floresiensis* at Liang Bua in Indonesia. Nature 532, 366–369 (2016). 27027286 10.1038/nature17179

[13] T. Sutikna, M. W. Tocheri, J. T. Faith, Jatmiko, R. A. Due, H. J. M. Meijer, E. W. Saptomo, R. G. Roberts, The spatio-temporal distribution of archaeological and faunal finds at Liang Bua (Flores, Indonesia) in light of the revised chronology for *Homo floresiensis*. J. Hum. Evol. 124, 52–74 (2018). 30173885 10.1016/j.jhevol.2018.07.001

[14] D. C. D’Amore, R. J. Blumenschine, Komodo monitor (*Varanus komodoensis*) feeding behavior and dental function reflected through tooth marks on bone surfaces, and the application to ziphodont paleobiology and the application to ziphodont paleobiology. Paleobiology 35, 525–552 (2009).

[15] G. Piga, M. D. Baró, I. G. Escobal, D. Gonçalves, C. Makhoul, A. Amarante, A. Malgosa, S. Enzo, S. Garroni, A structural approach in the study of bones: Fossil and burnt bones at nanosize scale. Appl. Phys. A 122, 1031 (2016).

[16] P. Shipman, G. Foster, M. Schoeninger, Burnt bones and teeth: An experimental study of color, morphology, crystal structure and shrinkage. J. Archaeol. Sci. 11, 307–325 (1984).

[17] T. Sutikna, “New archaeological research at Liang Bua on the Island of Flores: Implications for the extinction of *Homo floresiensis* and the arrival of *Homo sapiens* in Eastern Indonesia,” thesis, University of Wollongong, Wollongong, Australia (2016).

[18] M. W. Morley, P. Goldberg, T. Sutikna, M. W. Tocheri, L. C. Prinsloo, Jatmiko, E. W. Saptomo, S. Wasisto, R. G. Roberts, Initial micromorphological results from Liang Bua, Flores (Indonesia): Site formation processes and hominin activities at the type locality of *Homo floresiensis*. J. Archaeol. Sci. 77, 125–142 (2017).

[19] E. G. Veatch, “The zooarchaeology and taphonomy of small mammal remains at Liang Bua, Flores, Indonesia,” thesis, Emory University, Atlanta, GA (2021).

[20] C. Tennie, J. Call, M. Tomasello, Ratcheting up the ratchet: On the evolution of cumulative culture. Philos. Trans. R. Soc. London Ser. B. Biol. Sci. 364, 2405–2415 (2009). 19620111 10.1098/rstb.2009.0052 PMC2865079

[21] M. W. Tocheri, E. G. Veatch, Jatmiko, E. W. Saptomo, T. Sutikna, “*Homo floresiensis*”, in *The Oxford Handbook of Early Southeast Asia*, C. F. W. Higman, N. C. Kim, Eds. (Oxford Academic, 2022).

[22] F. Berna, P. Goldberg, L. K. Horwitz, J. Brink, S. Holt, M. Bamford, M. Chazan, Microstratigraphic evidence of in situ fire in the Acheulean strata of Wonderwerk Cave, Northern Cape province, South Africa. Proc. Natl. Acad. Sci. U.S.A. 109, E1215–E1220 (2012). 22474385 10.1073/pnas.1117620109 PMC3356665

[23] A. Brumm, G. M. Jensen, G. D. van den Bergh, M. J. Morwood, I. Kurniawan, F. Aziz, M. Storey, Hominins on Flores, Indonesia, by one million years ago. Nature 464, 748–752 (2010). 20237472 10.1038/nature08844

[24] A. Brumm, G. D. V. D. Bergh, M. Storey, I. Kurniawan, V. Brent, E. Setiyabudi, R. Grün, W. Mark, Age and context of the oldest known hominin fossils from Flores. Nature 534, 249–253 (2016). 27279222 10.1038/nature17663

[25] G. D. van den Bergh, Y. Kaifu, I. Kurniawan, R. T. Kono, A. Brumm, E. Setiyabudi, F. Aziz, M. J. Morwood, G. D. V. D. Bergh, Y. Kaifu, I. Kurniawan, R. T. Kono, A. Brumm, E. Setiyabudi, F. Aziz, M. J. Morwood, *Homo floresiensis*-like fossils from the early Middle Pleistocene of Flores. Nature 534, 245–248 (2016). 27279221 10.1038/nature17999

[26] M. Domínguez-Rodrigo, Hunting and scavenging by early humans: The state of the debate. J. World Prehist. 16, 1–54 (2002).

[27] M. Domínguez-Rodrigo, H. T. Bunn, J. Yravedra, A critical re-evaluation of bone surface modification models for inferring fossil hominin and carnivore interactions through a multivariate approach: Application to the FLK Zinj archaeofaunal assemblage (Olduvai Gorge, Tanzania). Quat. Int. 322–323, 32–43 (2014).

[28] M. C. Pante, R. J. Blumenschine, S. D. Capaldo, R. S. Scott, Validation of bone surface modification models for inferring fossil hominin and carnivore feeding interactions, with reapplication to FLK 22, Olduvai Gorge, Tanzania. J. Hum. Evol. 63, 395–407 (2012). 22192864 10.1016/j.jhevol.2011.09.002

[29] B. L. Pobiner, The zooarchaeology and paleoecology of early hominin scavenging. Evol. Anthropol. 29, 68–82 (2020). 32108400 10.1002/evan.21824

[30] S. Z. Goldenberg, G. Wittemyer, Elephant behavior toward the dead: A review and insights from field observations. Primates 61, 119–128 (2020). 31713106 10.1007/s10329-019-00766-5

[31] N. Sharma, S. S. Pokharel, S. Kohshima, R. Sukumar, Behavioural responses of free-ranging Asian elephants (*Elephas maximus*) towards dying and dead conspecifics. Primates 61, 129–138 (2020). 31428950 10.1007/s10329-019-00739-8

[32] E. Hadjisterkotis, D. S. Reese, Considerations on the potential use of cliffs and caves by the extinct endemic late pleistocene hippopotami and elephants of Cyprus. Eur. J. Wildl. Res. 54, 122–133 (2008).

[33] B. J. Schoville, E. Otárola-Castillo, A model of hunter-gatherer skeletal element transport: The effect of prey body size, carriers, and distance. J. Hum. Evol. 73, 1–14 (2014). 25059517 10.1016/j.jhevol.2014.06.004

[34] W. Auffenberg, *The Behavioral Ecology of the Komodo Monitor* (University Press of Florida, 1981).

[35] B. G. Fry, S. Wroe, W. Teeuwisse, M. J. P. van Osch, K. Moreno, J. Ingle, C. McHenry, T. Ferrara, P. Clausen, H. Scheib, K. L. Winter, L. Greisman, K. Roelants, L. van der Weerd, C. J. Clemente, E. Giannakis, W. C. Hodgson, S. Luz, P. Martelli, K. Krishnasamy, E. Kochva, H. F. Kwok, D. Scanlon, J. Karas, D. M. Citron, E. J. C. Goldstein, J. E. Mcnaughtan, J. A. Norman, A central role for venom in predation by *Varanus komodoensis* (Komodo Dragon) and the extinct giant *Varanus* (Megalania) *priscus*. Proc. Natl. Acad. Sci. U.S.A. 106, 8969–8974 (2009). 19451641 10.1073/pnas.0810883106 PMC2690028

[36] B. Pobiner, L. Dumouchel, J. Parkinson, A new semi-quantitative method for coding carnivore chewing damage with an application to modern african lion-damaged bones. Palaios 35, 302–315 (2020).

[37] A. J. Sutcliffe, Spotted hyaena: Crusher, gnawer, digester and collector of bones. Nature 227, 1110–1113 (1970). 5451104 10.1038/2271110a0

[38] W. L. Abler, The serrated teeth of tyrannosaurid dinosaurs, and biting structures in other animals. Paleobiology 18, 161–183 (1992).

[39] C. Ciofi, The Komodo dragon. Sci. Am. 280, 84–91 (1999).10232964

[40] G. Haynes, Late quaternary proboscidean sites in africa and eurasia with possible or probable evidence for hominin involvement. Quaternary 5, 18 (2022).

[41] G. Haynes, K. Krasinski, Butchering marks on bones of *Loxodonta africana* (African savanna elephant): Implications for interpreting marks on fossil proboscidean bones. J. Archaeol. Sci. Rep. 37, 102957 (2021).

[42] K. E. Krasinski, “Broken Bones and Cutmarks: Taphonomic Analyses and Implications for the Peopling of North America,” thesis, University of Nevada, Reno, NV (2010).

[43] K. D. Lupo, D. N. Schmitt, When bigger is not better: The economics of hunting megafauna and its implications for Plio-Pleistocene hunter-gatherers. J. Anthropol. Archaeol. 44, 185–197 (2016).

[44] S. Bunimovitz, R. Barkai, Ancient bones and modern myths: Ninth millennium BC hippopotamus hunters at Akrotiri Aetokremmos, Cyprus? J. Mediterr. Archaeol. 9, 85–96 (1996).

[45] M. Moore, T. Sutikna, M. Morwood, A. Brumm, Continuities in stone flaking technology at Liang Bua, Flores, Indonesia. J. Hum. Evol. 57, 503–526 (2009). 19361835 10.1016/j.jhevol.2008.10.006

[46] M. W. Moore, A. Brumm, “*Homo floresiensis* and the African Oldowan”, in *Interdisciplinary Approaches to the Oldowan*, E. Hovers, D. R. Braun, Eds. (Springer, 2008), pp. 61–69.

[47] M. W. Moore, A. Brumm, Stone artifacts and hominins in island Southeast Asia: New insights from Flores, eastern Indonesia. J. Hum. Evol. 52, 85–102 (2007). 17069874 10.1016/j.jhevol.2006.08.002

[48] R. Wrangham, R. Carmody, Human adaptation to the control of fire. Evol. Anthropol. 19, 187–199 (2010).

[49] D. M. Bramble, D. E. Lieberman, Endurance running and the evolution of *Homo*. Nature 432, 345–352 (2004). 15549097 10.1038/nature03052

[50] W. L. Jungers, W. E. H. Harcourt-Smith, R. E. Wunderlich, M. W. Tocheri, S. G. Larson, T. Sutikna, R. A. Due, M. J. Morwood, The foot of *Homo floresiensis*. Nature 459, 81–84 (2009). 19424155 10.1038/nature07989

[51] S. G. Larson, W. L. Jungers, M. J. Morwood, T. Sutikna, E. W. Saptomo, R. A. D. , T. Djubiantono, *Homo floresiensis* and the evolution of the hominin shoulder. J. Hum. Evol. 53, 718–731 (2007). 17692894 10.1016/j.jhevol.2007.06.003

[52] S. G. Larson, W. L. Jungers, M. W. Tocheri, C. M. Orr, M. J. Morwood, T. Sutikna, R. D. Awe, T. Djubiantono, Descriptions of the upper limb skeleton of *Homo floresiensis*. J. Hum. Evol. 57, 555–570 (2009). 19056103 10.1016/j.jhevol.2008.06.007

[53] C. M. Orr, M. W. Tocheri, S. E. Burnett, R. A. Due, E. W. Saptomo, T. Sutikna, S. Wasisto, M. J. Morwood, W. L. Jungers, R. D. Awe, E. W. Saptomo, T. Sutikna, Jatmiko, S. Wasisto, M. J. Morwood, W. L. Jungers, New wrist bones of *Homo floresiensis* from Liang Bua (Flores, Indonesia). J. Hum. Evol. 64, 109–129 (2013). 23290261 10.1016/j.jhevol.2012.10.003

[54] N. T. Roach, M. Venkadesan, M. J. Rainbow, D. E. Lieberman, Elastic energy storage in the shoulder and the evolution of high-speed throwing in *Homo*. Nature 498, 483–486 (2013). 23803849 10.1038/nature12267 PMC3785139

[55] M. W. Tocheri, C. M. Orr, S. G. Larson, T. Sutikna, Jatmiko, E. W. Saptomo, R. A. Due, T. Djubiantono, M. J. Morwood, W. L. Jungers, The primitive wrist of *Homo floresiensis* and its implications for hominin evolution. Science 317, 1743–1745 (2007). 17885135 10.1126/science.1147143

[56] W. L. Jungers, S. G. Larson, W. Harcourt-Smith, M. J. Morwood, T. Sutikna, R. A. Due, T. Djubiantono, Descriptions of the lower limb skeleton of *Homo floresiensis*. J. Hum. Evol. 57, 538–554 (2009). 19062072 10.1016/j.jhevol.2008.08.014

[57] M. J. Morwood, W. L. Jungers, Conclusions: Implications of the Liang Bua excavations for hominin evolution and biogeography. J. Hum. Evol. 57, 640–648 (2009). 19913680 10.1016/j.jhevol.2009.08.003

[58] M. C. Pante, M. V. Muttart, T. L. Keevil, R. J. Blumenschine, J. K. Njau, S. R. Merritt, A new high-resolution 3-D quantitative method for identifying bone surface modifications with implications for the Early Stone Age archaeological record. J. Hum. Evol. 102, 1–11 (2017). 28012460 10.1016/j.jhevol.2016.10.002

[59] M. J. Morwood, T. Sutikna, E. W. Saptomo, D. R. Hobbs, K. E. Westaway, Jatmiko, D. R. Hobbs, K. E. Westaway, Preface: Research at Liang Bua, Flores, Indonesia. J. Hum. Evol. 57, 437–449 (2009). 19733385 10.1016/j.jhevol.2009.07.003

[60] P. Villa, E. Mahieu, Breakage pattern of human long bones. J. Hum. Evol. 21, 27–48 (1991).

[61] R. L. Lyman, Quantitative units and terminology in zooarchaeology. Am. Antiq. 59, 36–71 (1994).

[62] R. J. Blumenschine, C. W. Marean, S. D. Capaldo, Blind tests of inter-analyst correspondence and accuracy in the identification of cut marks, percussion marks, and carnivore tooth marks on bone surfaces. J. Archaeol. Sci. 23, 493–507 (1996).

[63] R. J. Blumenschine, M. M. Selvaggio, Percussion marks on bone surfaces as a new diagnostic of hominid behaviour. Nature 333, 763–765 (1988).

[64] D. R. Braun, M. Pante, W. Archer, Cut marks on bone surfaces: Influences on variation in the form of traces of ancient behaviour. Interface Focus 6, 20160006 (2016). 27274806 10.1098/rsfs.2016.0006 PMC4843629

[65] M. Domínguez-Rodrigo, S. de Juana, A. B. Galán, M. Rodríguez, A new protocol to differentiate trampling marks from butchery cut marks. J. Archaeol. Sci. 36, 2643–2654 (2009).

[66] A. K. Behrensmeyer, Taphonomic and ecologic information from bone weathering. Paleobiology 4, 150–162 (1978).

[67] S. E. Rhodes, M. J. Walker, A. López-Jiménez, M. López-Martínez, M. Haber-Uriarte, Y. Fernandez-Jalvo, M. Chazan, Fire in the Early Palaeolithic: Evidence from burnt small mammal bones at Cueva Negra del Estrecho del Río Quípar, Murcia, Spain. J. Archaeol. Sci. Rep. 9, 427–436 (2016).

[68] Y. Fernández-Jalvo, P. Andrews, *Atlas of Taphonomic Identifications* (Springer Science+Business Media, 2016).

[69] J. C. Thompson, The impact of post-depositional processes on bone surface modification frequencies: A corrective strategy and its application to the Loiyangalani site, Serengeti Plain, Tanzania. J. Taphon. 3, 67–89 (2005).

[70] T. L. Keevil, “Inferring Early Stone Age Tool Technology And Raw Material From Cut Mark Micromorphology Using High-Resolution 3-D Scanning With Applications To Middle Bed II, Olduvai Gorge, Tanzania,” thesis, Colorado State University, Fort Collins, CO (2018).

[71] M. Friendly, M. Sigal, Graphical methods for multivariate linear models in psychological research: An R tutorial. Quant. Method. Psychol. 13, 20–45 (2017).

[72] B. D. Ripley, *Pattern Recognition and Neural Networks* (Cambridge Univ. Press, 1996).

[73] W. N. Venables, B. D. Ripley, *Modern Applied Statistics with S.* (Springer, ed. 4, 2002).

[74] QGIS, QGIS Geographic Information System (QGIS Association; 2022); http://qgis.org/.

[75] G. Haynes, P. Wojtal, Weathering stages of proboscidean bones: Relevance for zooarchaeological analysis. J. Archaeol. Method Theory 30, 495–535 (2023).

[76] A. A. E. van der Geer, G. D. van den Bergh, G. A. Lyras, U. W. Prasetyo, R. A. Due, E. Setiyabudi, H. Drinia, The effect of area and isolation on insular dwarf proboscideans. J. Biogeogr. 43, 1656–1666 (2016).

[77] E. G. Veatch, I. M. A. Julianto, Jatmiko, T. Sutikna, M. W. Tocheri, Prey body size generates bias for human and avian agents: Cautions for interpreting small game assemblages. J. Archaeol. Sci. 160, 105883 (2023).

[78] D. A. Byers, A. Ugan, Should we expect large game specialization in the late Pleistocene? An optimal foraging perspective on early Paleoindian prey choice. J. Archaeol. Sci. 32, 1624–1640 (2005).

[79] K. D. Lupo, J. M. Fancher, D. N. Schmitt, The taphonomy of resource intensification: Zooarchaeological implications of resource scarcity among Bofi and Aka forest foragers. J. Archaeol. Method Theory 20, 420–447 (2013).

