# Supplementary material for: Taphonomic analysis at Liang Bua reveals the behavioral and technological capabilities of Homo floresiensis
Source: Sci Adv. 2026 Jul 3;12(27):eaeb7219. doi: 10.1126/sciadv.aeb7219 (PMC13330864; doi:10.1126/sciadv.aeb7219)
Supplement: Supplementary file 1 — Supplementary Text Figs. S1 to S7 Tables S1 to S6 Legends for data S1 to S5 Legend for movie S1 References [file sciadv.aeb7219_sm.pdf]

Science Advances
AAAS

Supplementary Materials for

Taphonomic analysis at Liang Bua reveals the behavioral and technological capabilities of *Homo floresiensis*

E. Grace Veatch *et al.*

Corresponding author: E. Grace Veatch, elizabeth.veatch@gmail.com

Sci. Adv. **12**, eaeb7219 (2026)
DOI: 10.1126/sciadv.aeb7219

The PDF file includes:

Supplementary Text
Figs. S1 to S7
Tables S1 to S6
Legends for data S1 to S5
Legend for movie S1
References

Other Supplementary Material for this manuscript includes the following:

Data S1 to S5
Movie S1

Supplementary Text

<u>Quantitative analysis of Komodo tooth scores and cutmarks from Liang Bua</u>

3D Analysis

The 3D quadradic discriminant analysis (QDA) using the resubstitution model classified the experimental groups with 84% correct classification (Table S2). When applied to a sample of unknown marks (n = 55) from the *Stegodon* bones, the model identified 30 KTM, nine CM, and two trample marks with a posterior score of 0.7 or greater, leaving 14 marks indeterminate (Table S3). After reviewing each mark for qualitative features commonly associated with cutmarks (i.e., shoulder effect, etc.) and Komodo dragon tooth marks (i.e., posterior fan, etc.), a total of 40 KTM, seven CM, and eight indeterminate marks were identified (Data S3).

It's important to note the limitations and potential issues impacting our analysis. First, the comparative marks used in this analysis were collected on goat and bovid bones, which may or may not adequately reflect how cutmarks and Komodo tooth marks manifest on elephant bones. For example, another experimental study involving the butchery of elephant long bones and ribs did describe the resulting marks as quite shallow (*41*). However, depth was not measured in this study, so it is unclear whether cutmarks on elephant carcasses are quantifiably shallower than on bovids. In addition, our cutmark comparative sample is restricted to marks created on long bone shafts and does not include marks on other elements, such as scapula, vertebrae, pelves, or ribs, but the Komodo dragon experimental sample does include these elements as well as long bone shafts and epiphyses (Data S1). There are also qualitative features of Komodo dragon tooth marks that were not captured by the quantitative analysis, such as the microstriation fan or the presence/absence of internal microstriations.

3D QDA Re-substitution Model:

<u>Prior probabilities of groups:</u>

CM	KTM	Trample
0.33	0.33	0.33

<u>Group means reported based on raw data:</u>

	**CM**	**KTM**	**Trample**
**VOL**	187352659.40	18793649.35	137447379.10
**MD**	83.44	42.02	87.34

**MEAN**	29.39	16.01	26.34
**ML**	8521.19	3538.22	8079.05
**MW**	414.44	349.90	724.90
**MDP**	76.24	34.03	76.15
**A**	23611.89	7372.89	24066.20
**W**	333.95	370.45	493.48
**RA**	2.99	1.31	4.41
**ANG**	125.77	156.18	143.80
**RAD**	521.78	869.20	1962.71

Percent correctly classified:
0.8545455

Type II MANOVA Tests: Wilks test statistic

	Df	test stat	approx F	num Df	den Df	Pr(>F)
GROUP	2	0.32023	41.286	22	1184	< 2.2e-16 ***

2D Analysis

The 2D QDA using the resubstitution model classified the experimental groups with 74% correct classification – 10% less than the 3D model (Table S4). The canonical scores between groups are statistically significant (p <0.0001), with CM having longer and thinner dimensions compared to KTM. Almost all the variables included in the analysis are statistically significant from one another ($p < 0.05$) with the exception of perimeter (Fig. S8). Overall, the 2D analysis confirms that Komodo dragon tooth marks tend to be shorter and wider than cutmarks, as was shown in the 3D analysis. While depth was not captured in the 2D analysis, the method showed to be successful in separating the two groups.

When applied to a sample of unknown marks (n = 51) from the *Stegodon* bones, the model identified 22 KTM and 18 CM with a posterior score of 0.7 or greater, leaving 11 marks indeterminate. After reviewing each mark for qualitative features commonly associated with cutmarks (i.e., shoulder effect, etc.) and Komodo dragon tooth marks (i.e., posterior fan, etc.), a total of 35 KTM, 14 CM, and two indeterminate marks were identified (Data S5).

2D QDA Re-substitution Model:

Prior probabilities of groups:

CM	KTM
0.5	0.5

Group means reported based on raw data:

	**CM**	**KTM**
**Area**	0.85	1.17
**Perim**	11.83	9.39
**Length**	4.70	3.79
**Width**	0.23	0.40
**Angle**	49.63	68.44
**Circ**	0.11	0.21
**AR**	26.24	13.89
**Round**	0.06	0.13

Percent correctly classified:
0.7421875

Type II MANOVA Tests: Wilks test statistic

	Df	test stat	approx F	num Df	den Df	Pr(>F)
GROUP	1	0.69776	6.4433	8	119	6.031e-07 ***

Additional taphonomic results

Weathering stages indicate that all elements experienced some damage due to surface exposure prior to burial (Table S3). Eighty-three percent of mature/adult bones retain a stage 1 or 2 of surface damage indicating burial in ~1–4 years and 13% with stage 3 surface damage indicating burial in ~4–8+ years (*66*). Comparatively, 62% of bones with subadult features were likely buried in ~1–4 years and 35% in ~4–8+ years. However, Hayes and Wojtal (*75*) note that the surfaces of proboscidean bones weather differently depending on age and should be taken into consideration when estimating time of burial. Unfortunately, this publication was not available at the time of data collection. Still, these patterns suggest that all *Stegodon* elements were affected by some weathering damage with a range of burial rates estimated from as little as ~1 to 8 years.

Provenance Section

Since 2001, archaeological excavations at Liang Bua have been carried out under formal collaborations between Pusat Penelitian Arkeologi Nasional (currently Badan Riset dan Inovasi Nasional [BRIN] in Jakarta, Indonesia) and University of New England, University of

Wollongong, Smithsonian Institution's National Museum of Natural History, and Lakehead University. Our study sample consisted of non-dental skeletal remains of *Stegodon* from Liang Bua, which we randomly sampled from Sectors I, III, IV, VII, XI, XIV, XV, XVII, XXI, XXII, XXIII. Sectors I, III, and IV represent 3 x 3 m areas excavated during field seasons that took place between 2001 and 2003 (*59*). Each of the other Sectors represents a 2 x 2 m area excavated during field seasons that took place between 2003 and 2011 (*59*, *12*, and *13*). Sectors were excavated in 10 cm intervals (referred to as spits) while following stratigraphic layers. Findings that were visible upon excavation were plotted in three dimensions. Sediments in each spit were hand sieved followed by wet sieving with 2 mm mesh. Recovered findings were cleaned, sorted, cataloged in bags, and transported to BRIN for curation and further study. Associated date ranges for the specimens sampled in this study were previously published by Thomas Sutikna et al. (*12*). All specimens are currently housed at BRIN facilities in Indonesia (Jakarta and Cibinong) and access to these collections is managed by BRIN through Pusat Penelitian Arkeometri and Organisasi Riset Arkeologi, Bahasa, dan Sastra. Validation of authenticity was done by BRIN.

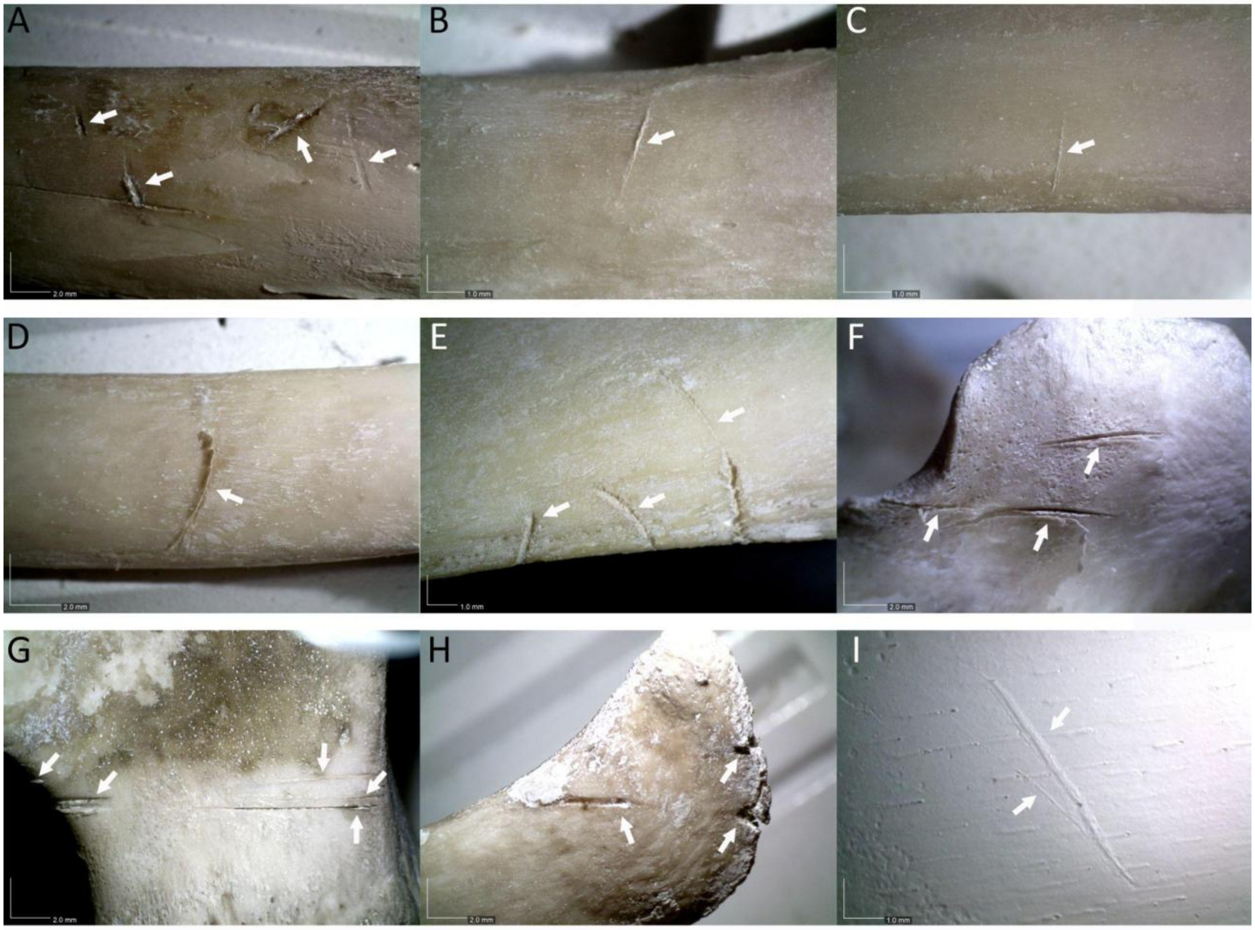

Fig. S1.
Examples of Komodo dragon tooth marks from experiments conducted at Zoo Atlanta. **(A)** Four relatively wide scores on a rib bone. **(B)** and **(C)** two small shallow isolated striations on rib bones. **(D)** and **(E)** curved striations on a rib and scapula, respectively. **(F)** and **(G)** deep parallel striations that resemble cutmarks on vertebrae. **(H)** A relatively wide striation and two pits located on the edge of the transverse process on a lumbar vertebra. **(I)** A mold impression of a shallow mark located on a humerus with a branching score and parallel microstriations resembling a shoulder effect.

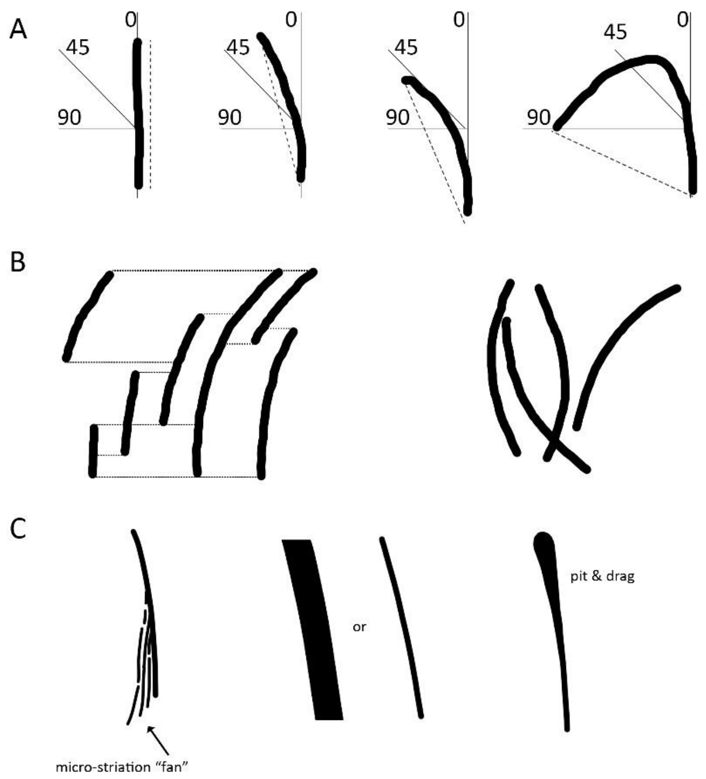

Fig. S2.
Types of tooth marks made by Komodo dragons. **(A)** Variation in mark curvature and **(B)** clustering combinations (redrawn from D'Amore and Blumenschine *(14)* reproduced with permission). **(C)** Variation of mark form identified using 3D profilometry. Some marks exhibit a microstriation "fan" that is formed by contact with the serrated posterior edge of the tooth. Marks also vary in width ranging from relatively shallow and wide to narrow and deep (narrow mean value = 221.3 µm; wide mean value = 391.9 µm). Depth also varies with some marks showing a deep pit-like depression that gradually becomes shallow across the score. For additional descriptions of Komodo dragon tooth mark shape, cluster patterns, and angles, see D'Amore and Blumenschine (*14*).

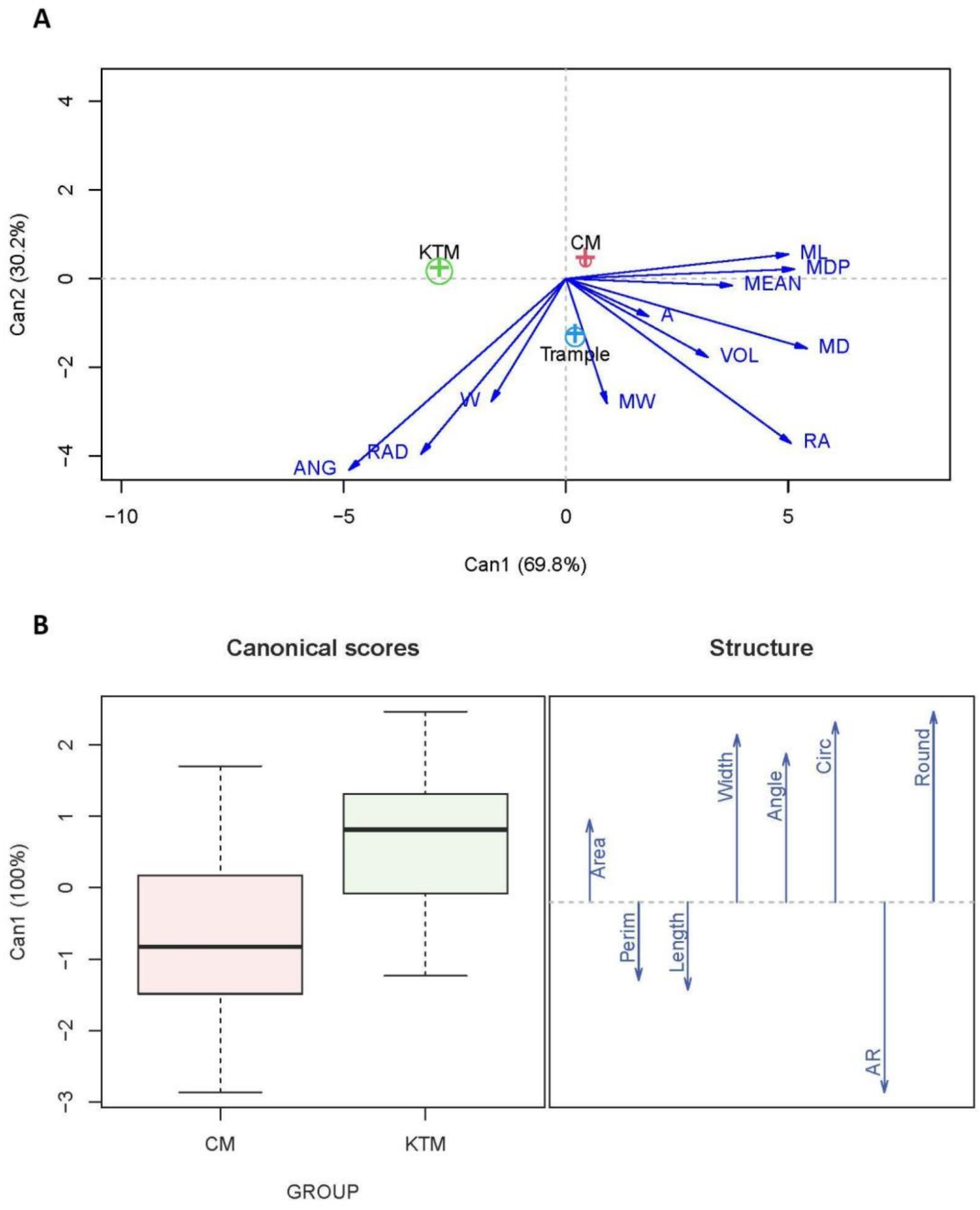

Fig. S3.
Results from the **(A)** 3D and **(B)** 2D canonical discriminant model showing the center and range of scores according to group and the loading of each variable.

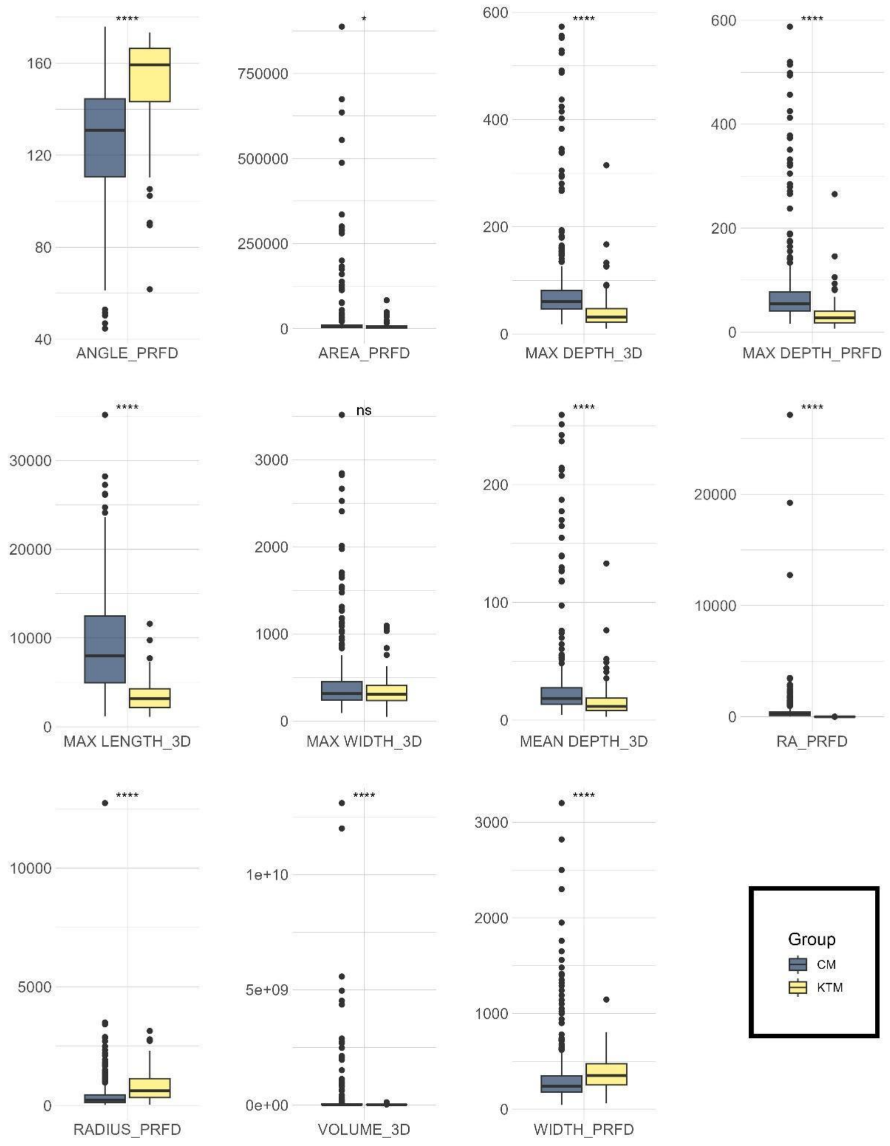

Fig. S4.
Boxplots showing a comparison between experimentally generated cutmarks (CM) and Komodo tooth marks (KTM) with variables used in the 3D QDA. ns = not significant; * = 0.05; ** = 0.01; *** = 0.001; **** = 0.0001. Center line = median; upper and lower hinges = 1st and 3rd quartiles (25th and 75th percentiles); whiskers = 1.5 times the interquartile range; points = outliers.

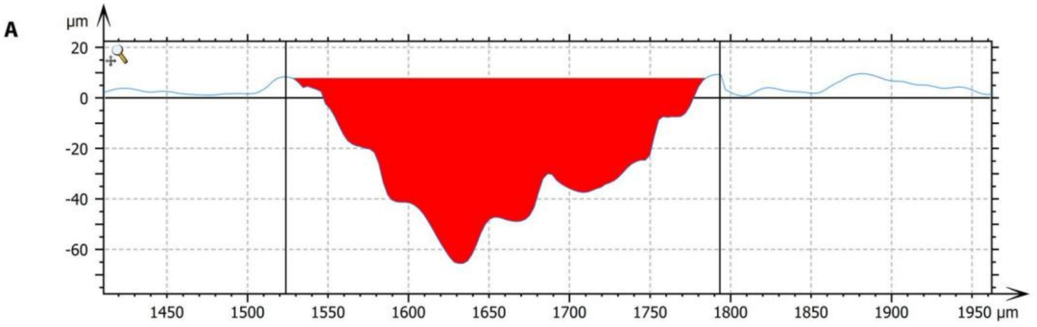

Parameters	Value	Unit
Maximum depth	73.9211	µm
Area of the hole	9928.45	µm²

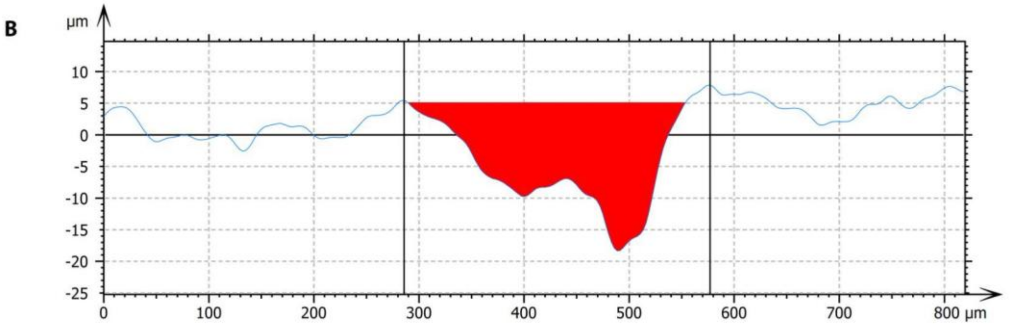

Parameters	Value	Unit
Maximum depth	23.6965	µm
Area of the hole	3082.66	µm²

Fig. S5

Profiles of experimentally generated cutmark **(A)** and a Komodo dragon tooth mark **(B)** at the deepest point along the mark.

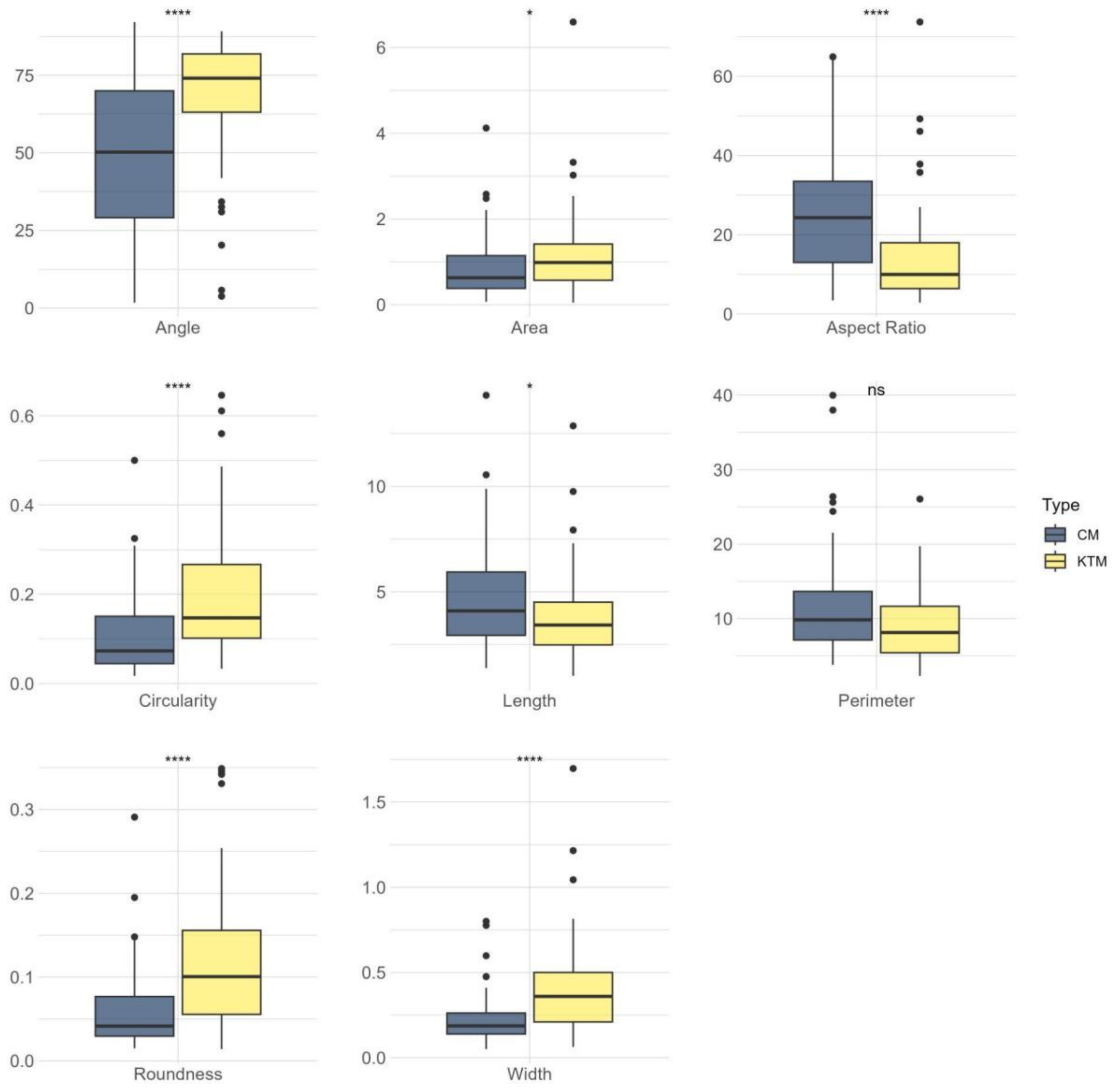

Fig. S6.
Boxplots showing a comparison between experimentally generated cutmarks (CM) and Komodo tooth marks (KTM) with variables used in the 2D QDA. ns = not significant; * = 0.05; ** = 0.01; *** = 0.001; **** = 0.0001. Center line = median; upper and lower hinges = 1st and 3rd quartiles (25th and 75th percentiles); whiskers = 1.5 times the interquartile range; points = outliers.

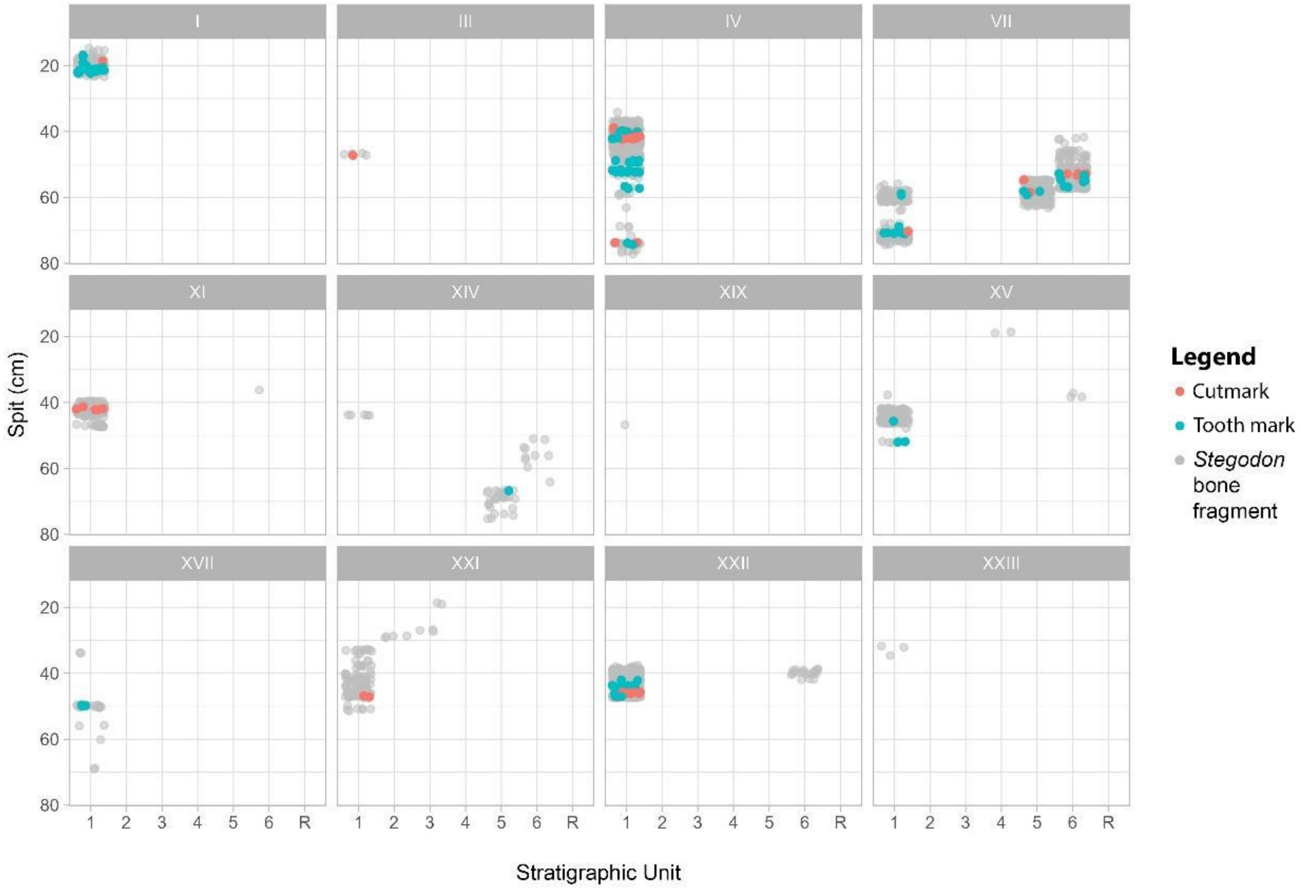

Fig. S7.
Jitter plots of *Stegodon* skeletal elements sampled from Liang Bua according to the recovery location (Sector, spit (depth), and stratigraphic unit). Bones with cutmarks and Komodo dragon toothmarks reported here are shown in pink and teal, respectively. Units 1 and 2 contain *in situ* sediments associated with *H. floresiensis* while units 3–8A from Sectors VII, XI, XIV, XV, XXI, and XXII contain *Stegodon* elements along with eroded and reworked sediments that originally derive from units 1 and/or 2 (See Methods). R denotes bones recovered from wall cleanings.

Table S1.
Summary results of marks from controlled feeding experiments comparing this study and D'Amore and Blumenschine (*14*).

	This Study	D'Amore and Blumenschine (2009)
n carcasses	1	11
Mean marks per carcass (range)	192	78.8 (0-330)
Mean marks per element (range)	7.4 (1-55)	5.5 (1-16)
Marks in clusters	70.83%	32%
Internal microstriations	14.06%	*NA*
External microstriations	1.04%	*NA*
Fan presence	7.29%	*NA*
Curvature estimates:		
Straight	59.46%	46%
curved <45*	27.57%	30%
curved 45-90*	5.41%	10%
curved >90*	1.08%	0%

Table S2.

Classifications of groups from the 3D training QDA model using re-substitution shown in number and percentage.

	Actual (values)				Actual (percentages)		
Predicted	CM	KTM	Trample	Predicted	CM	KTM	Trample
CM	345	2	24	CM	0.86	0.03	0.185
KTM	15	69	3	KTM	0.04	0.96	0.023
Trample	43	1	103	Trample	0.11	0.01	0.792

Table S3.

Summary of taphonomic processes and categories observed in the *Stegodon* assemblage at Liang Bua (listed as percentages of the study sample).

Degree	Root Etching	Exfoliation*	Corrosion	Sedimentary Abrasion	Sheen	Smoothing	Insect Activity	Bacterial
Absent	95.2	34.9	75.9	98.4	97.5	89.6	97	98.7
Present	4.1	64.4	23.3	0.9	1.7	9.7	2.3	0.6
Indet.	0.7	0.7	0.8	0.7	0.7	0.7	0.7	0.7

Scale	Surface Visibility
0	0.3
10%	0.7
20%	2.5
30%	6
40%	5.3
50%	9.5
60%	7.7
70%	17.4
80%	20.6
90%	29.4
100%	0.6

Category	Rib Circumference
< 1/2	7.8
> 1/2	74.4
Complete	17.9

Category	Fossilization
None	0.6
Light	59.3
Moderate	33.3
Heavy	6.8

Category	Break
Postdepositional	60
Recent	28
Indet.	12

Stage	Oxide Staining	Weathering Adult	Weathering Subadult
Stage 0	51.1	0	0
Stage 1	36.9	46	43.5
Stage 2	11.1	37.2	18.8
Stage 3	0.9	13.3	34.8
Stage 4	-	3.5	2.3
Stage 5	-	0	0

Description	Color
White	1.2
Light Beige	83.9
Beige	13.8
Grey	0.6
Multi-colored	0.3
Indet.	0.3

* high presence of exfoliation is likely due to how juveile proboscidean bones respond to weathering conditions compared to adults.

Table S4.

Classifications of groups from the 2D training QDA model using re-substitution shown in number and percentage.

	Actual (values)			Actual (percentages)	
Predicted	CM	KTM	Predicted	CM	KTM
CM	49	18	CM	0.765	0.281
KTM	15	46	KTM	0.234	0.718

Table S5.

Summary of mark frequencies and frequencies of skeletal elements with marks attributed to agents with high confidence. QDA contains marks included in the 3D and 2D analyses. High confidence marks were not included in the 3D and 2D analysis.

		Number of Marked Bones			% Cutmarked Bone per Element Frequency	% Tooth Marked Bone per Element Frequency	Number of Marks					
							CM		KTM		Indeterminate	
Element	Element Frequency	CM	KTM	**Total**			QDA	High Confidence	QDA	High Confidence	QDA	**Total**
Cranial	33	1		**1**	**3.0**	**0.0**	1					**1**
Cranial Fragment	1011		2	**2**	**0.0**	**0.2**			2			**2**
Femur	4		1	**1**	**0.0**	**25.0**			1	14		**15**
Fibula	7		1	**1**	**0.0**	**14.3**			7			**7**
Innominate	5	1		**1**	**20.0**	**0.0**	1				2	**1**
Long-Bone Fragment	373	5	5	**10**	**1.3**	**1.3**	4	13	10			**27**
Metapodial	3		1	**1**	**0.0**	**33.3**			1			**1**
Non-ID Fragment	189		1	**1**	**0.0**	**0.5**			3	3		**6**
Phalanx	6	1		**1**	**16.7**	**0.0**		3				**3**
Radius	5	1	1	**2**	**20.0**	**20.0**	1		1	2		**4**
Rib	510	9	23	**32**	**1.8**	**4.5**	13	14	47	5	4	**79**
Scapula	69			**0**	**0.0**	**0.0**					3	**0**
Sternal	3		1	**1**	**0.0**	**33.3**			2			**2**
Stylohyoid	1	1		**1**	**100.0**	**0.0**		3				**3**
Thoracic	5	1		**1**	**20.0**	**0.0**	1					**1**
Ulna	5		1	**1**	**0.0**	**20.0**			1	1		**2**
**Total**	**2229**	**20**	**37**	**57**	**0.9**	**1.7**	**21**	**33**	**75**	**25**	**9**	**129**

Table S6.

Prey rank using the Prey Choice Model to estimate post-encounter returns (Ei/hi). All values are sourced from citations within Lupo and Schmitt (*43*) except for *Stegodon* and *Papagomys.*

Species	English Name	Body Weight (kg)[b]	Edible Body Weight (kg)[b]	Handling Costs (mns) Pursuit Time	Butchery Time	Total	Success Rate	Post-Encounter Returns	Rank
Synceru scaffer	Buffalo	533	320	180	481	661	0.5	18880.5	1
Sylvicapra grimmia	Bush Duiker	18.5	15	13	49	62	0.8	15329	2
Equus quagga	Zebra	239	132	60	302	362	0.5	14767.9	3
Pedetes capensis	Springhare	3.5	3	10	12	22	0.8	10407.3	4
Raphicerus campestris	Steenbok	11.5	9.2	18	23	41	0.72	10178.3	5
Otocyon megalotis	Bat-eared Fox	3.6	3	16	12	28	0.8	8177.1	6
Connochaetes taurinus	Wildebeest	227	114	544	44	588	0.5	7503.1	7
Tragelaphus strepisceros	Kudu	215	108	40	481	521	0.4	6567.1	8
Papagomys armandvillei	Flores Giant Rat	2.5[f]	2[h]	31[j]	10.6[j]	41.6	1 g	6237.2	9
Aepyceros melampus	Impala	51	33	150	26	176	0.5	5737.5	10
Alcelaphus buselaphus	Hartebeest	130	65	544	44	588	0.5	3880.1	11
Loxodonta africanus	African Elephant	4104	1724	2282	5175	7457	0.2	3606.6	12
Stegodon florensis	Flores Stegodon	1703[e]	357[g]	2282[i]	1345.4[k]	3627.4	0.2	3076.1	13
Phacochoerus africanus	Warthog	70	46	600	30	630	0.5	2891.4	14
Tauritragus oryx	Eland	337	219	1893	540	2433	0.4	2700.4	15
Oryx gazella	Gemsbok	215	108	474	481	955	0.3	2687	16
Stegodon florensis insularis (Adult)	Dwarfed Flores Stegodon	570[e]	239.4[g]	1141[i]	450.3[k]	1591.3	0.2	2346.9	17
Giraffa camelopardis	Giraffe	983	590	4800	770	5570	0.2	1563.4	18
Stegodon florensis insularis (Juvenile)	Dwarfed Flores Stegodon	125.4[d]	52.7[g]	1141[i]	99.1[k]	1240.1	0.2	662.6	19
Stegodon florensis insularis (Calf)	Dwarfed Flores Stegodon	27.4[c]	11.5[g]	1141[i]	21.6[k]	1162.6	0.2	154.2	20

[a] Raw data used to estimate handling costs and success rates from Appendix A in Lupo and Schmitt (*43*) unless otherwise specified.

[b] Values are tabulated in Lupo and Schmitt (*43*) (See citations within) unless otherwise specified.

[c] Based on the proportion of African elephant calf (200 kg) to adult (4104 kg) weight values.

[d] Based on the proportion of African elephant juvenile (900 kg) to adult (4104 kg) weight values.

[e] Estimated from van der Greer (*76*)

[f] Value from Veatch et al. (*77*) (specimen ID #6).

[g] Edible proportion of Proboscidean carcasses (0.42) derived from Byers and Ugan (*78*) reported in Lupo and Schmitt (*43*).

[h] Edible weight proportion (0.8) estimated from Lupo et al (*79*) based on experimental butchery of springhare with similar body size proportions as the Flores giant rat.

[i] Pursuit time of 2282 from Lupo and Schmitt (*43*) averaged from ethnographic accounts for African elephant captures. This time is applied to the large *Stegodon florensis* and cut in half (1141) for the smaller *Stegodon florensis insularis.* While pursuit time is unknown in this context, we are assuming the pursuit time would be less given the smaller body size of the animal.

[j] Unpublished value from ethnoarchaeological study presented in Veatch et al. (*77*).

[k] Butchery time is estimated as a proportion of butchery time:body weight ratios of the African elephant (0.79) used in Lupo and Schmitt (*43*).

Data S1. (Separate File)

Summary of Komodo dragon tooth scores on goat bones generated from captive feeding experiment at Zoo ATL.

Data S2. (Separate File)

Raw profilometry data of comparative Komodo dragon tooth scores and marks from the Liang Bua *Stegodon* assemblage.

Data S3 (Separate File)

Input and output data from the 3D quadratic discriminant analysis (QDA), including the QDA classification (class) and our final mark assessment (Final ID) with accompanying notes.

Data S4 (Separate File)

Raw 2D data of comparative Komodo dragon tooth scores, cutmarks, and marks from the Liang Bua *Stegodon* assemblage.

Data S5 (Separate File)

Input and output data from the 2D quadratic discriminant analysis (QDA), including the QDA classification (class) and our final mark assessment (Final ID) with accompanying notes.

Movie S1.

Video of the captive Komodo dragon at Zoo Atlanta consuming a dressed goat carcass.

REFERENCES

1. P. Brown, T. Sutikna, M. J. Morwood, R. P. Soejono, Jatmiko, E. W. Saptomo, R. A. Due, A new small-bodied hominin from the Late Pleistocene of Flores, Indonesia. *Nature* **431**, 1055–1061 (2004).

2. M. J. Morwood, P. Brown, Jatmiko, T. Sutikna, E. W. Saptomo, K. E. Westaway, R. A. Due, R. G. Roberts, T. Maeda, S. Wasisto, T. Djubiantono, Further evidence for small-bodied hominins from the Late Pleistocene of Flores, Indonesia. *Nature* **437**, 1012–1017 (2005).

3. M. J. Morwood, R. P. Soejono, R. G. Roberts, T. Sutikna, C. S. M. Turney, K. E. Westaway, W. J. Rink, J.-x. Zhao, G. D. van den Bergh, R. A. Due, D. R. Hobbs, M. W. Moore, M. I. Bird, L. K. Fifield, Archaeology and age of a new hominin from Flores in eastern Indonesia. *Nature* **431**, 1087–1091 (2004).

4. G. D. van den Bergh, H. J. M. Meijer, R. A. Due, M. J. Morwood, K. Szabó, L. W. van den Hoek Ostende, T. Sutikna, E. W. Saptomo, P. J. Piper, K. M. Dobney, The Liang Bua faunal remains: A 95 k.yr. sequence from Flores, East Indonesia. *J. Hum. Evol.* **57**, 527–537 (2009).

5. G. D. van den Bergh, R. D. Awe, M. J. Morwood, T. Sutikna, Jatmiko, E. W. Saptomo, The youngest *Stegodon* remains in Southeast Asia from the Late Pleistocene archaeological site Liang Bua, Flores, Indonesia. *Quat. Int.* **182**, 16–48 (2008).

6. D. Falk, C. Hildebolt, K. Smith, M. Morwood, T. Sutikna, P. Brown, Jatmiko, E. W. Saptomo, B. Brunsden, F. Prior, The brain of LB1, *Homo floresiensis*. *Science* **308**, 242–245 (2005).

7. R. Barkai, J. Rosell, R. Blasco, A. Gopher, Fire for a reason: Barbecue at Middle Pleistocene Qesem Cave, Israel. *Curr. Anthropol.* **58**, S314–S328 (2017).

8. R. Wrangham, Control of fire in the paleolithic: Evaluating the cooking hypothesis. *Curr. Anthropol.* **58**, S303–S313 (2017).

9. K. Isler, C. P. van Schaik, How humans evolved large brains: Comparative evidence. *Evol. Anthropol.* **23**, 65–75 (2014).

10. R. J. Blumenschine, Carcass consumption sequences and the archaeological distinction of scavenging and hunting. *J. Hum. Evol.* **15**, 639–659 (1986).

11. R. J. Blumenschine, K. A. Prassack, C. D. Kreger, M. C. Pante, Carnivore tooth-marks, microbial bioerosion, and the invalidation of Domınguez-Rodrigo and Barba's (2006) test of Oldowan hominin scavenging behavior. *J. Hum. Evol.* **53**, 420–426 (2007).

12. T. Sutikna, M. W. Tocheri, M. J. Morwood, E. W. Saptomo, Jatmiko, R. A. Due, S. Wasisto, K. E. Westaway, M. Aubert, B. Li, J.-x. Zhao, M. Storey, B. V. Alloway, M. W. Morley, H. J. M. Meijer, G. D. van den Bergh, R. Grün, A. Dosseto, A. Brumm, W. L. Jungers, R. G. Roberts, Revised stratigraphy and chronology for *Homo floresiensis* at Liang Bua in Indonesia. *Nature* **532**, 366–369 (2016).

13. T. Sutikna, M. W. Tocheri, J. T. Faith, Jatmiko, R. A. Due, H. J. M. Meijer, E. W. Saptomo, R. G. Roberts, The spatio-temporal distribution of archaeological and faunal finds at Liang Bua (Flores, Indonesia) in light of the revised chronology for *Homo floresiensis*. *J. Hum. Evol.* **124**, 52–74 (2018).

14. D. C. D'Amore, R. J. Blumenschine, Komodo monitor (*Varanus komodoensis*) feeding behavior and dental function reflected through tooth marks on bone surfaces, and the application to ziphodont paleobiology and the application to ziphodont paleobiology. *Paleobiology* **35**, 525–552 (2009).

15. G. Piga, M. D. Baró, I. G. Escobal, D. Gonçalves, C. Makhoul, A. Amarante, A. Malgosa, S. Enzo, S. Garroni, A structural approach in the study of bones: Fossil and burnt bones at nanosize scale. *Appl. Phys. A* **122**, 1031 (2016).

16. P. Shipman, G. Foster, M. Schoeninger, Burnt bones and teeth: An experimental study of color, morphology, crystal structure and shrinkage. *J. Archaeol. Sci.* **11**, 307–325 (1984).

17. T. Sutikna, "New archaeological research at Liang Bua on the Island of Flores: Implications for the extinction of *Homo floresiensis* and the arrival of *Homo sapiens* in Eastern Indonesia," thesis, University of Wollongong, Wollongong, Australia (2016).

18. M. W. Morley, P. Goldberg, T. Sutikna, M. W. Tocheri, L. C. Prinsloo, Jatmiko, E. W. Saptomo, S. Wasisto, R. G. Roberts, Initial micromorphological results from Liang Bua, Flores (Indonesia): Site formation processes and hominin activities at the type locality of *Homo floresiensis*. *J. Archaeol. Sci.* **77**, 125–142 (2017).

19. E. G. Veatch, "The zooarchaeology and taphonomy of small mammal remains at Liang Bua, Flores, Indonesia," thesis, Emory University, Atlanta, GA (2021).

20. C. Tennie, J. Call, M. Tomasello, Ratcheting up the ratchet: On the evolution of cumulative culture. *Philos. Trans. R. Soc. London Ser. B. Biol. Sci.* **364**, 2405–2415 (2009).

21. M. W. Tocheri, E. G. Veatch, Jatmiko, E. W. Saptomo, T. Sutikna, "*Homo floresiensis*", in *The Oxford Handbook of Early Southeast Asia*, C. F. W. Higman, N. C. Kim, Eds. (Oxford Academic, 2022).

22. F. Berna, P. Goldberg, L. K. Horwitz, J. Brink, S. Holt, M. Bamford, M. Chazan, Microstratigraphic evidence of in situ fire in the Acheulean strata of Wonderwerk Cave, Northern Cape province, South Africa. *Proc. Natl. Acad. Sci. U.S.A.* **109**, E1215–E1220 (2012).

23. A. Brumm, G. M. Jensen, G. D. van den Bergh, M. J. Morwood, I. Kurniawan, F. Aziz, M. Storey, Hominins on Flores, Indonesia, by one million years ago. *Nature* **464**, 748–752 (2010).

24. A. Brumm, G. D. V. D. Bergh, M. Storey, I. Kurniawan, V. Brent, E. Setiyabudi, R. Grün, W. Mark, Age and context of the oldest known hominin fossils from Flores. *Nature* **534**, 249–253 (2016).

25. G. D. van den Bergh, Y. Kaifu, I. Kurniawan, R. T. Kono, A. Brumm, E. Setiyabudi, F. Aziz, M. J. Morwood, G. D. V. D. Bergh, Y. Kaifu, I. Kurniawan, R. T. Kono, A. Brumm, E. Setiyabudi, F. Aziz, M. J. Morwood, *Homo floresiensis*-like fossils from the early Middle Pleistocene of Flores. *Nature* **534**, 245–248 (2016).

26. M. Domínguez-Rodrigo, Hunting and scavenging by early humans: The state of the debate. *J. World Prehist.* **16**, 1–54 (2002).

27. M. Domínguez-Rodrigo, H. T. Bunn, J. Yravedra, A critical re-evaluation of bone surface modification models for inferring fossil hominin and carnivore interactions through a multivariate approach: Application to the FLK Zinj archaeofaunal assemblage (Olduvai Gorge, Tanzania). *Quat. Int.* **322–323**, 32–43 (2014).

28. M. C. Pante, R. J. Blumenschine, S. D. Capaldo, R. S. Scott, Validation of bone surface modification models for inferring fossil hominin and carnivore feeding interactions, with reapplication to FLK 22, Olduvai Gorge, Tanzania. *J. Hum. Evol.* **63**, 395–407 (2012).

29. B. L. Pobiner, The zooarchaeology and paleoecology of early hominin scavenging. *Evol. Anthropol.* **29**, 68–82 (2020).

30. S. Z. Goldenberg, G. Wittemyer, Elephant behavior toward the dead: A review and insights from field observations. *Primates* **61**, 119–128 (2020).

31. N. Sharma, S. S. Pokharel, S. Kohshima, R. Sukumar, Behavioural responses of free-ranging Asian elephants (*Elephas maximus*) towards dying and dead conspecifics. *Primates* **61**, 129–138 (2020).

32. E. Hadjisterkotis, D. S. Reese, Considerations on the potential use of cliffs and caves by the extinct endemic late pleistocene hippopotami and elephants of Cyprus. *Eur. J. Wildl. Res.* **54**, 122–133 (2008).

33. B. J. Schoville, E. Otárola-Castillo, A model of hunter-gatherer skeletal element transport: The effect of prey body size, carriers, and distance. *J. Hum. Evol.* **73**, 1–14 (2014).

34. W. Auffenberg, *The Behavioral Ecology of the Komodo Monitor* (University Press of Florida, 1981).

35. B. G. Fry, S. Wroe, W. Teeuwisse, M. J. P. van Osch, K. Moreno, J. Ingle, C. McHenry, T. Ferrara, P. Clausen, H. Scheib, K. L. Winter, L. Greisman, K. Roelants, L. van der Weerd, C. J. Clemente, E. Giannakis, W. C. Hodgson, S. Luz, P. Martelli, K. Krishnasamy, E. Kochva, H. F. Kwok, D. Scanlon, J. Karas, D. M. Citron, E. J. C. Goldstein, J. E. Mcnaughtan, J. A. Norman, A central role for venom in predation by *Varanus komodoensis* (Komodo Dragon)

and the extinct giant *Varanus* (Megalania) *priscus*. *Proc. Natl. Acad. Sci. U.S.A.* **106**, 8969–8974 (2009).

36. B. Pobiner, L. Dumouchel, J. Parkinson, A new semi-quantitative method for coding carnivore chewing damage with an application to modern african lion-damaged bones. *Palaios* **35**, 302–315 (2020).

37. A. J. Sutcliffe, Spotted hyaena: Crusher, gnawer, digester and collector of bones. *Nature* **227**, 1110–1113 (1970).

38. W. L. Abler, The serrated teeth of tyrannosaurid dinosaurs, and biting structures in other animals. *Paleobiology* **18**, 161–183 (1992).

39. C. Ciofi, The Komodo dragon. *Sci. Am.* **280**, 84–91 (1999).

40. G. Haynes, Late quaternary proboscidean sites in africa and eurasia with possible or probable evidence for hominin involvement. *Quaternary* **5**, 18 (2022).

41. G. Haynes, K. Krasinski, Butchering marks on bones of *Loxodonta africana* (African savanna elephant): Implications for interpreting marks on fossil proboscidean bones. *J. Archaeol. Sci. Rep.* **37**, 102957 (2021).

42. K. E. Krasinski, "Broken Bones and Cutmarks: Taphonomic Analyses and Implications for the Peopling of North America," thesis, University of Nevada, Reno, NV (2010).

43. K. D. Lupo, D. N. Schmitt, When bigger is not better: The economics of hunting megafauna and its implications for Plio-Pleistocene hunter-gatherers. *J. Anthropol. Archaeol.* **44**, 185–197 (2016).

44. S. Bunimovitz, R. Barkai, Ancient bones and modern myths: Ninth millennium BC hippopotamus hunters at Akrotiri Aetokremmos, Cyprus? *J. Mediterr. Archaeol.* **9**, 85–96 (1996).

45. M. Moore, T. Sutikna, M. Morwood, A. Brumm, Continuities in stone flaking technology at Liang Bua, Flores, Indonesia. *J. Hum. Evol.* **57**, 503–526 (2009).

46. M. W. Moore, A. Brumm, "*Homo floresiensis* and the African Oldowan", in *Interdisciplinary Approaches to the Oldowan*, E. Hovers, D. R. Braun, Eds. (Springer, 2008), pp. 61–69.

47. M. W. Moore, A. Brumm, Stone artifacts and hominins in island Southeast Asia: New insights from Flores, eastern Indonesia. *J. Hum. Evol.* **52**, 85–102 (2007).

48. R. Wrangham, R. Carmody, Human adaptation to the control of fire. *Evol. Anthropol.* **19**, 187–199 (2010).

49. D. M. Bramble, D. E. Lieberman, Endurance running and the evolution of *Homo*. *Nature* **432**, 345–352 (2004).

50. W. L. Jungers, W. E. H. Harcourt-Smith, R. E. Wunderlich, M. W. Tocheri, S. G. Larson, T. Sutikna, R. A. Due, M. J. Morwood, The foot of *Homo floresiensis*. *Nature* **459**, 81–84 (2009).

51. S. G. Larson, W. L. Jungers, M. J. Morwood, T. Sutikna, E. W. Saptomo, R. A. D. , T. Djubiantono, *Homo floresiensis* and the evolution of the hominin shoulder. *J. Hum. Evol.* **53**, 718–731 (2007).

52. S. G. Larson, W. L. Jungers, M. W. Tocheri, C. M. Orr, M. J. Morwood, T. Sutikna, R. D. Awe, T. Djubiantono, Descriptions of the upper limb skeleton of *Homo floresiensis*. *J. Hum. Evol.* **57**, 555–570 (2009).

53. C. M. Orr, M. W. Tocheri, S. E. Burnett, R. A. Due, E. W. Saptomo, T. Sutikna, S. Wasisto, M. J. Morwood, W. L. Jungers, R. D. Awe, E. W. Saptomo, T. Sutikna, Jatmiko, S. Wasisto, M. J. Morwood, W. L. Jungers, New wrist bones of *Homo floresiensis* from Liang Bua (Flores, Indonesia). *J. Hum. Evol.* **64**, 109–129 (2013).

54. N. T. Roach, M. Venkadesan, M. J. Rainbow, D. E. Lieberman, Elastic energy storage in the shoulder and the evolution of high-speed throwing in *Homo*. *Nature* **498**, 483–486 (2013).

55. M. W. Tocheri, C. M. Orr, S. G. Larson, T. Sutikna, Jatmiko, E. W. Saptomo, R. A. Due, T. Djubiantono, M. J. Morwood, W. L. Jungers, The primitive wrist of *Homo floresiensis* and its implications for hominin evolution. *Science* **317**, 1743–1745 (2007).

56. W. L. Jungers, S. G. Larson, W. Harcourt-Smith, M. J. Morwood, T. Sutikna, R. A. Due, T. Djubiantono, Descriptions of the lower limb skeleton of *Homo floresiensis*. *J. Hum. Evol.* **57**, 538–554 (2009).

57. M. J. Morwood, W. L. Jungers, Conclusions: Implications of the Liang Bua excavations for hominin evolution and biogeography. *J. Hum. Evol.* **57**, 640–648 (2009).

58. M. C. Pante, M. V. Muttart, T. L. Keevil, R. J. Blumenschine, J. K. Njau, S. R. Merritt, A new high-resolution 3-D quantitative method for identifying bone surface modifications with implications for the Early Stone Age archaeological record. *J. Hum. Evol.* **102**, 1–11 (2017).

59. M. J. Morwood, T. Sutikna, E. W. Saptomo, D. R. Hobbs, K. E. Westaway, Jatmiko, D. R. Hobbs, K. E. Westaway, Preface: Research at Liang Bua, Flores, Indonesia. *J. Hum. Evol.* **57**, 437–449 (2009).

60. P. Villa, E. Mahieu, Breakage pattern of human long bones. *J. Hum. Evol.* **21**, 27–48 (1991).

61. R. L. Lyman, Quantitative units and terminology in zooarchaeology. *Am. Antiq.* **59**, 36–71 (1994).

62. R. J. Blumenschine, C. W. Marean, S. D. Capaldo, Blind tests of inter-analyst correspondence and accuracy in the identification of cut marks, percussion marks, and carnivore tooth marks on bone surfaces. *J. Archaeol. Sci.* **23**, 493–507 (1996).

63. R. J. Blumenschine, M. M. Selvaggio, Percussion marks on bone surfaces as a new diagnostic of hominid behaviour. *Nature* **333**, 763–765 (1988).

64. D. R. Braun, M. Pante, W. Archer, Cut marks on bone surfaces: Influences on variation in the form of traces of ancient behaviour. *Interface Focus* **6**, 20160006 (2016).

65. M. Domínguez-Rodrigo, S. de Juana, A. B. Galán, M. Rodríguez, A new protocol to differentiate trampling marks from butchery cut marks. *J. Archaeol. Sci.* **36**, 2643–2654 (2009).

66. A. K. Behrensmeyer, Taphonomic and ecologic information from bone weathering. *Paleobiology* **4**, 150–162 (1978).

67. S. E. Rhodes, M. J. Walker, A. López-Jiménez, M. López-Martínez, M. Haber-Uriarte, Y. Fernandez-Jalvo, M. Chazan, Fire in the Early Palaeolithic: Evidence from burnt small mammal bones at Cueva Negra del Estrecho del Río Quípar, Murcia, Spain. *J. Archaeol. Sci. Rep.* **9**, 427–436 (2016).

68. Y. Fernández-Jalvo, P. Andrews, *Atlas of Taphonomic Identifications* (Springer Science+Business Media, 2016).

69. J. C. Thompson, The impact of post-depositional processes on bone surface modification frequencies: A corrective strategy and its application to the Loiyangalani site, Serengeti Plain, Tanzania. *J. Taphon.* **3**, 67–89 (2005).

70. T. L. Keevil, "Inferring Early Stone Age Tool Technology And Raw Material From Cut Mark Micromorphology Using High-Resolution 3-D Scanning With Applications To Middle Bed II, Olduvai Gorge, Tanzania," thesis, Colorado State University, Fort Collins, CO (2018).

71. M. Friendly, M. Sigal, Graphical methods for multivariate linear models in psychological research: An R tutorial. *Quant. Method. Psychol.* **13**, 20–45 (2017).

72. B. D. Ripley, *Pattern Recognition and Neural Networks* (Cambridge Univ. Press, 1996).

73. W. N. Venables, B. D. Ripley, *Modern Applied Statistics with S.* (Springer, ed. 4, 2002).

74. QGIS, QGIS Geographic Information System (QGIS Association; 2022); http://qgis.org/.

75. G. Haynes, P. Wojtal, Weathering stages of proboscidean bones: Relevance for zooarchaeological analysis. *J. Archaeol. Method Theory* **30**, 495–535 (2023).

76. A. A. E. van der Geer, G. D. van den Bergh, G. A. Lyras, U. W. Prasetyo, R. A. Due, E. Setiyabudi, H. Drinia, The effect of area and isolation on insular dwarf proboscideans. *J. Biogeogr.* **43**, 1656–1666 (2016).

77. E. G. Veatch, I. M. A. Julianto, Jatmiko, T. Sutikna, M. W. Tocheri, Prey body size generates bias for human and avian agents: Cautions for interpreting small game assemblages. *J. Archaeol. Sci.* **160**, 105883 (2023).

78. D. A. Byers, A. Ugan, Should we expect large game specialization in the late Pleistocene? An optimal foraging perspective on early Paleoindian prey choice. *J. Archaeol. Sci.* **32**, 1624–1640 (2005).

79. K. D. Lupo, J. M. Fancher, D. N. Schmitt, The taphonomy of resource intensification: Zooarchaeological implications of resource scarcity among Bofi and Aka forest foragers. *J. Archaeol. Method Theory* **20**, 420–447 (2013).
